# Engineering copper and copper-based materials for a post-antibiotic era

**DOI:** 10.3389/fbioe.2025.1644362

**Published:** 2025-08-06

**Authors:** Yingxian Wang, Tongqiang Wen, Fuchao Mao, Shaozhe Yang, Qingwei Zhang, Xiuhong Fu, Chongkai Zhai, Hewei Zhang

**Affiliations:** ^1^ School of Mechanical and Electrical Engineering, Luoyang Polytechnic, Luoyang, Henan, China; ^2^ College of Food and Drugs, Luoyang Polytechnic, Luoyang, Henan, China; ^3^ Animal Diseases and Public Health Engineering Research Center of Henan Province, Luoyang, Henan, China; ^4^ The Geographical Indication Medicines and Life Health Engineering Research Center of Henan Province, Luoyang, Henan, China; ^5^ Luoyang Key Laboratory of Diagnosis and Immunoprophylaxis of Animal Viral Diseases, Luoyang, Henan, China; ^6^ Henan Luohe Central Hospital, Luohe, Henan, China

**Keywords:** copper-based materials, nanotechnology, surface engineering, antimicrobial activity, applications

## Abstract

In recent years, the emergence of multidrug-resistant bacteria and the frequent outbreaks of novel viral pathogens have intensified the demand for novel, efficient, and low-toxicity antimicrobial materials. Copper and copper-based materials, owing to their broad-spectrum and potent antimicrobial properties, have attracted increasing attention across diverse fields, including medicine, agriculture, and environmental science. This review provides a comprehensive overview of the development history, antimicrobial and antiviral mechanisms, fabrication techniques, and bioactive characteristics of copper and its derivatives. It further highlights their current applications in healthcare, public infrastructure, food processing, textiles, and modern agriculture. Challenges associated with material stability, cytotoxicity and environmental safety, are critically discussed. Finally, future perspectives are proposed, emphasizing advances in material synthesis, the development of stable nano-coatings, controlled release strategies, low-toxicity and low-resistance formulations, establishment of standardized pharmacological and toxicological evaluation systems, drug delivery applications, and copper pollution control. This review aims to inform future efforts in overcoming the current limitations of copper-based antimicrobials and supporting their potential future integration into applications across medicine, public health, environmental protection, and agricultural innovation, contingent upon resolving current translational and regulatory challenges.

## 1 Introduction

Microbial infections remain a persistent and escalating threat to global public health and food security, driven by the rapid emergence of multidrug-resistant (MDR) bacterial strains and novel viral pathogens (Murray et al., 2022). According to the World Health Organization (WHO), antimicrobial resistance (AMR) could claim as many as 10 million lives annually by 2050 if current trends continue unchecked (Tang et al., 2023). The COVID-19 pandemic has further amplified the urgency of developing next-generation antimicrobial materials that are capable of interrupting surface-mediated transmission and curbing large-scale outbreaks (Van Doremalen et al., 2021).

Among candidate materials, copper and copper-based materials attract renewed scientific attention due to their intrinsic and broad-spectrum antimicrobial properties, long-standing use in medical and industrial contexts, and a relatively low propensity for resistance development (Grass et al., 2011). The antimicrobial efficacy of copper stems from its multifaceted mechanisms of action: copper ions are capable of disrupting bacterial cell membranes, interfering with intracellular enzyme activity, impairing metabolic function, inducing the generation of reactive oxygen species (ROS), protein dysfunction, and DNA degradation and inhibiting biofilm formation (Warnes et al., 2015). This multimodal activity renders copper fast-acting and potentially less prone to inducing microbial resistance compared to traditional antibiotics.

The application of nanotechnology has significantly enhanced the antimicrobial potential of copper by enabling the design of materials with enhanced surface-area-to-volume ratios, tunable ion release kinetics, and improved physicochemical stability. Notably, engineers have designed copper and copper-based nanoparticles with precise control over size, morphology, and surface chemistry, achieving significantly enhanced bactericidal and virucidal activity under physiologically relevant conditions (Ren et al., 2009; Yimeng et al., 2023). Simultaneously, green synthesis approaches using biological templates such as plant extracts, bacteria, and fungi have emerged as sustainable alternatives to conventional chemical synthesis. These eco-friendly methods reduce toxic byproducts and allow for better biocompatibility of the resultant nanoparticles (Priya et al., 2023). Furthermore, surface engineering strategies have enabled the creation of copper-based antimicrobial coatings that are suitable for high-touch surfaces in hospitals, public transportation, and food processing facilities. Techniques such as laser-induced forward transfer, electrochemical deposition, and plasma spraying have been successfully employed to create robust copper coatings on metals, polymers, and textiles (Gautam et al., 2024). Researchers are developing smart responsive systems that modulate copper ion release in response to pH, moisture, or bacterial load, promising improved efficacy and minimized adverse effects (Guo et al., 2024).

Despite these advances, several limitations continue to challenge the clinical and commercial adoption of copper-based antimicrobial technologies. The cytotoxicity of free copper ions remains a primary concern, particularly when used in biomedical implants or wound dressings. Researchers are exploring controlled-release formulations, such as encapsulated copper nanoparticles or biodegradable polymer matrices, to mitigate these effects (Pourmadadi et al., 2024). Another key challenge is the lack of international standards for evaluating the efficacy and long-term safety of copper-based antimicrobials. The scientific community urgently needs standardized assays that account for real-world conditions, including biofilm formation, fluid flow, and mixed microbial populations (Salah et al., 2021; Williams et al., 2023). Environmental sustainability is another critical consideration. Although copper is a naturally occurring element, excessive accumulation from industrial use may lead to ecological toxicity, particularly in aquatic and soil systems. Therefore, lifecycle assessments and ecological risk analyses should accompany the development of copper-based technologies (Samarajeewa et al., 2021; Vignardi et al., 2023).

Looking forward, copper’s integration into multifunctional composites and hybrid materials offers a promising avenue. For instance, copper–graphene and copper–zinc oxide heterostructures have demonstrated synergistic antimicrobial effects, combining membrane disruption with photothermal or photocatalytic activity (Lv R. et al., 2022). In wound healing, copper–hydrogel system provide not only antimicrobial protection but also pro-angiogenic effects, facilitating tissue regeneration (Geng et al., 2023; Zhou W. et al., 2020). In agriculture, copper-based nanofertilizers and pesticides show potential to reduce pathogen loads while enhancing plant growth, although regulatory hurdles remain significant (Su et al., 2024). The translation of these technologies into scalable products requires collaboration across disciplines, including microbiology, materials science, toxicology, and regulatory science. Open-access antimicrobial material databases and machine-learning-guided material design are poised to accelerate discovery pipelines (Zhao Y. et al., 2024).

In conclusion, copper and copper-based materials represent a promising component of the multifaceted approach needed to address the escalating problem of AMR. Their broad-spectrum activity, multimodal mechanisms, and adaptability across industrial and biomedical domains position them as strong candidates in the ongoing fight against infectious diseases. However, responsible innovation must address biosafety, environmental impact, and regulatory standardization to ensure the sustainable and equitable deployment of copper-based antimicrobial solutions. By leveraging modern materials science, synthetic biology, and systems-level design, the next-generation of copper-enabled antimicrobial systems may play a transformative role in addressing the growing burden of infectious diseases in a post-antibiotic era. This review aims to serve as a reference for accelerating innovation in the development and application of copper-based antimicrobial materials.

## 2 Tracing the antimicrobial legacy of copper: from ancient remedies to modern materials

**FIGURE 1:**
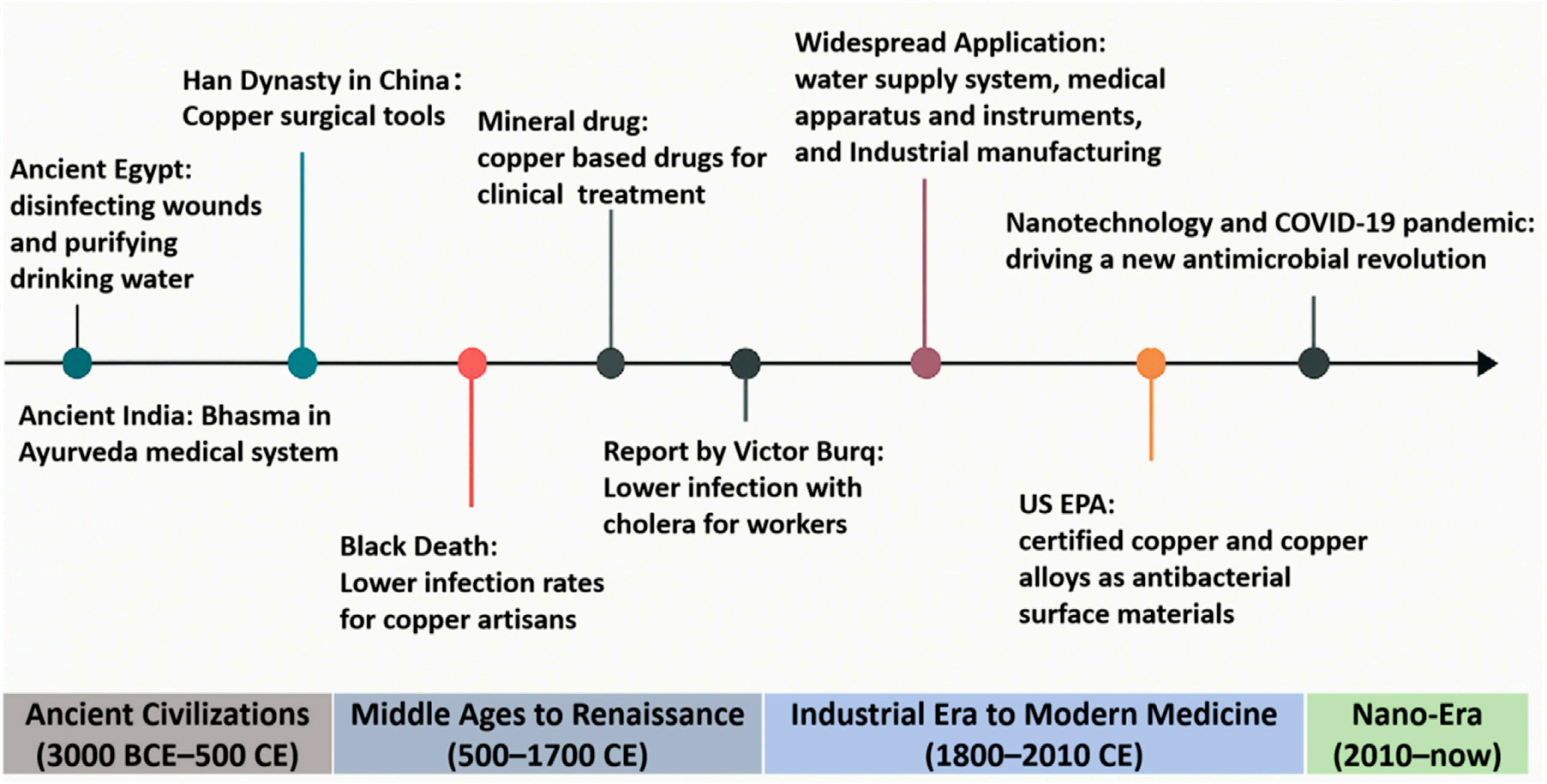
Timeline of the major historic events of copper and copper-based materials as an antimicrobial agent.

Copper, a naturally occurring transition metal, has been utilized by human civilizations for millennia due to its distinctive antimicrobial properties. Figure 1 illustrates major milestones in copper’s antimicrobial journey. The earliest records date back to around 3000 BCE, showing ancient Egyptians using copper compounds to treat wounds and sterilize drinking water. Copper containers were widely adopted to prolong the shelf-life of water and perishable food, minimizing microbial contamination and spoilage (Vincent et al., 2016).

Across ancient Greece and Rome, empirical observations reinforced copper’s preservative effects. Water stored in copper vessels remained fresher, prompting the use of copper in utensils, water pitchers, and even surgical tools (Pourmadadi et al., 2024). Hippocratic medical scripts from 400 BCE described the use of copper oxide and verdigris for the treatment of skin infections and ulcers (O’gorman and Humphreys, 2012). In both ancient China and India, copper served a similar function. Notably, the Ayurvedic medical tradition developed during the Vedic period (circa 1500–500 BCE) introduced Bhasma, a class of metallic-herbal nanomedicines produced by the incineration of copper and other metals with botanical ingredients. These particles, often in the 10–100 nm range, displayed remarkable biocompatibility and antimicrobial potential, presaging modern nanomedicine concepts (Adhikari, 2014; Sarkar and Das Mukhopadhyay, 2021). In China, archaeological finds from tombs in the Yellow River basin have unearthed copper surgical instruments dated to the Han Dynasty, further substantiating copper’s early medical utility (Wu, 2019).

Medieval European societies also recognized copper’s antimicrobial utility. Cooking in copper pots prevented spoilage, and by the Renaissance, physicians adopted copper-based tools and copper salts to treat wounds, noting reduced infection rates. In the 19th century, a new awareness of copper’s medical potency was spawned by the observation that copper workers appeared to be immune to cholera (Grass et al., 2011). These findings catalyzed broader industrial applications of copper in sanitation, water purification, and medical devices (Lin et al., 2021). In the contemporary context, copper and its alloys have found widespread applications in public health infrastructure. Since 2008, the U.S. Environmental Protection Agency (EPA) has officially classified copper alloys as antimicrobial surfaces, capable of killing over 99.9% of bacteria, including *Escherichia coli*, *Staphylococcus aureus*, and *Clostridium difficile*, within 2 hours of contact (Borkow and Gabbay, 2009). High-touch surfaces in hospitals and public settings, such as doorknobs, bed rails, and faucet handles, are now increasingly manufactured using copper or copper-containing alloys to reduce microbial persistence and cross-contamination (Butot et al., 2021).

Recent developments in alloy design have further optimized copper’s biomedical utility. Copper alloys, such as copper-infused stainless steel and copper-titanium composites, have been systematically evaluated for their biocompatibility and antimicrobial performance. Copper’s antimicrobial efficacy stems from its ability to release copper ions that damage bacterial cell components. Higher copper content generally leads to stronger antimicrobial action, but excessive copper can also cause cytotoxicity. The effectiveness of copper against bacteria is linked to its ability to damage cell walls, membranes, and DNA, often through the production of ROS (Mahmoudi et al., 2022). Clinical investigations revealed that incorporating copper surfaces into intensive care units can reduce hospital-acquired infection (HAI) rates by up to 58% (Arendsen et al., 2019). Interestingly, copper is less likely than antibiotics to induce resistance, a critical feature in the fight against multidrug-resistant organisms (MDROs) (Orta-Rivera et al., 2023). Clinical trials in hospital intensive care units (ICUs) showed a reduction of 83%–99.9% in pathogen burden on copper-coated surfaces of common objects in the ICU room (Glass et al., 2023).

The development of nanotechnology has significantly enhanced copper’s antimicrobial capabilities, leading to a renewed interest in its use for various applications (Crisan et al., 2021). These nanoscale materials are now incorporated into coatings for medical devices, implants, textiles, and even air and water filtration systems. Self-sterilizing copper nanocoatings can be applied to door handles, catheter tips, surgical trays, and implantable devices. These coatings ensure continuous antimicrobial ion release and maintain efficacy even after repeated microbial challenges. Additionally, copper nanoparticles are embedded into fabrics to create antimicrobial wound dressings, hospital linens, face masks, and protective clothing, which significantly reduce fomite-mediated disease transmission (Butler et al., 2023). Notably, copper nanoparticles exhibit potent efficacy against both Gram-positive and Gram-negative bacteria, fungi, and a wide range of enveloped and non-enveloped viruses (Ramos-Zúñiga et al., 2023). Copper nanoparticles usually work by generating ROS and oxidizing capsid proteins, inhibiting SARS-CoV-2, influenza H1N1, and norovirus on copper-embedded materials (Ha et al., 2022; Mosselhy et al., 2022). Copper’s broad-spectrum efficacy extends to fungal pathogens as well, with antifungal nanocoatings reducing *Candida* albicans adhesion on prosthetic surfaces by over 90% (Kadirvelu et al., 2024). A clinical trial involving copper-impregnated wound dressings reported accelerated epithelialization and reduced secondary infections in diabetic foot ulcers compared to silver-based alternatives (Borkow and Melamed, 2025).

Amid escalating global public health crises, marked by the emergence of antibiotic-resistant bacteria and the rapid evolution of viral pathogens, the advent of nanotechnology has catalyzed the development and deployment of nanocopper-based materials as next-generation antimicrobial agents. Nanocopper coatings are widely used across diverse sectors, including medical devices, food processing, public transportation, and educational facilities, due to their well-documented broad-spectrum antimicrobial activity (Mohammad and Ahmad, 2024). Additionally, nanocopper coatings and textiles reduce pathogen transmission in healthcare and public areas. They apply to high-contact surfaces like door handles and bed rails, creating self-sanitizing interfaces that kill microbes. Integrating nanocopper into textiles creates antimicrobial dressings and PPE, further mitigating pathogen spread (Bisht et al., 2022). Importantly, nanocopper materials have demonstrated strong inhibitory effects against MDR bacteria, including methicillin-resistant *S. aureus* (MRSA), representing a potential tool in the broader strategy to combat AMR, though further clinical validation is warranted (Wang et al., 2017a). These advances position nanocopper as a compelling component in the development of durable, broad-spectrum antimicrobial strategies, bridging material science with infectious disease control.

In summary, the antimicrobial journey of copper, from its empirical use in antiquity to its current status as a scientifically endorsed, nanotechnologically enhanced antimicrobial platform, highlights its notable versatility and promising translational potential in select antimicrobial contexts. Its enduring relevance is attributed to its multifaceted mechanisms of action, broad-spectrum efficacy, and relatively lower likelihood of resistance development compared to conventional antibiotics, although emerging copper-resistance mechanisms warrant continued monitoring. As the world grapples with antibiotic resistance and recurrent viral pandemics, copper and its nanostructured derivatives offer a valuable addition to the arsenal of antimicrobial strategies. Nonetheless, concerns surrounding cytotoxicity, environmental accumulation, and regulatory standardization must be addressed to fully realize copper’s potential as a safe and sustainable antimicrobial agent.

## 3 Multifaceted biocidal pathways: the antimicrobial arsenal of copper

**FIGURE 2:**
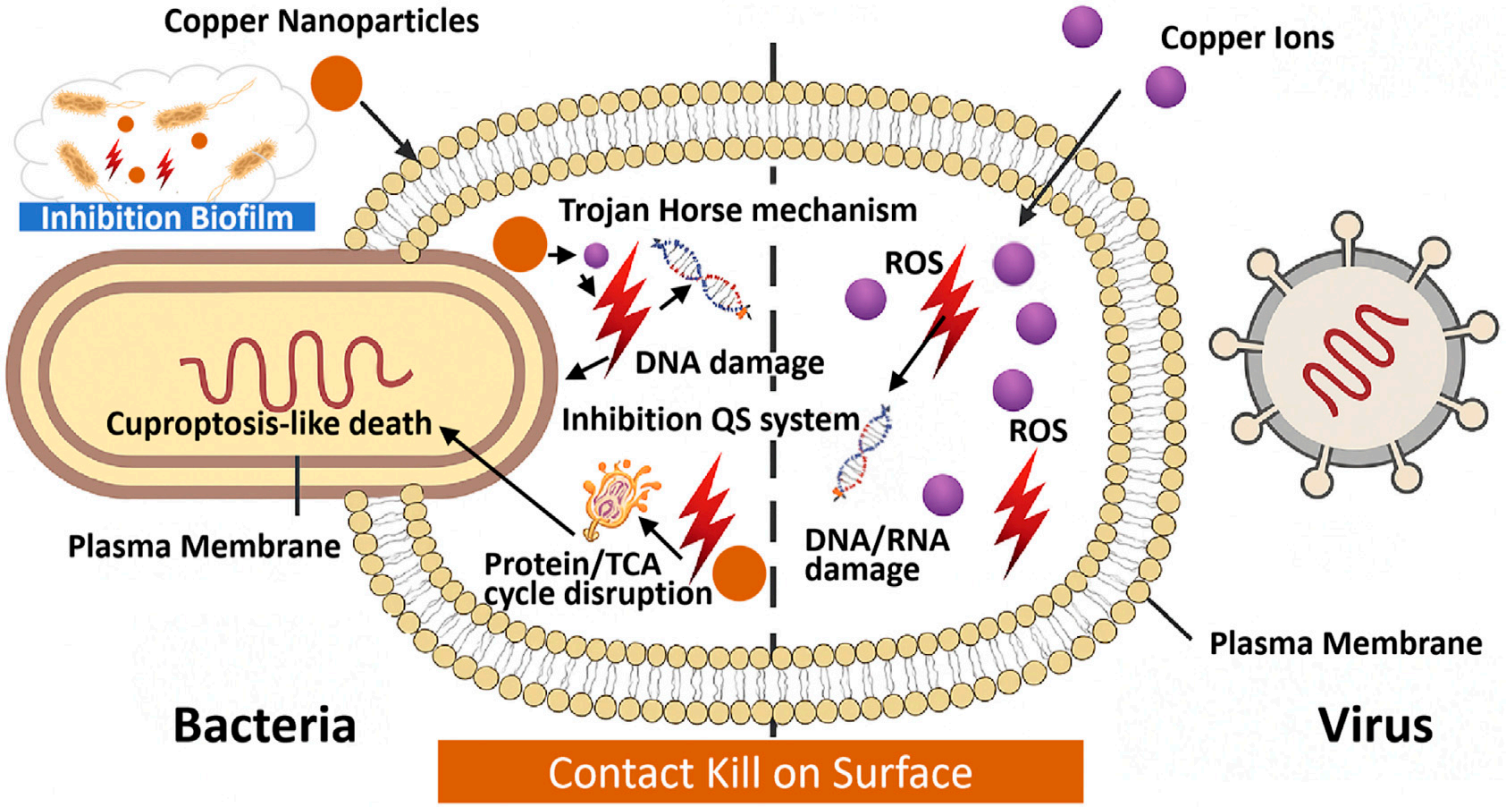
Schematic illustration of the mechanism of antimicrobial copper and copper-based materials. Conventional copper’s antimicrobial mechanisms. Copper ions bind to microbial membranes, causing depolarization and rupture. Intracellular copper ions induce ROS generation via Fenton-like reactions, leading to lipid peroxidation and protein dysfunction. Copper ions interact with DNA/RNA, causing strand breaks and inhibiting replication. Inhibition of biofilm formation through disruption of extracellular polymeric substances (EPS). Nanocopper’s antimicrobial mechanisms: High surface-area copper-based nanoparticles release copper ions rapidly, penetrating microbial membranes. Trojan horse mechanism: internalized copper-based nanoparticles release ions intracellularly, amplifying ROS production. ROS cause oxidative damage to lipids, proteins, and nucleic acids. Quorum sensing (QS) inhibition disrupts biofilm formation in *Pseudomonas aeruginosa*. Cuproptosis-like death via TCA cycle disruption.

Copper’s antimicrobial mechanisms are multifaceted, involving the generation of ROS, membrane depolarization, protein dysfunction, nucleic acid degradation, and inhibition of biofilm formation. In contrast to antibiotics, which typically target a single cellular pathway, copper exerts its effects through multiple and overlapping mechanisms. This multimodal action significantly reduces the likelihood of resistance development. A schematic overview of copper’s antimicrobial mechanisms is presented in Figure 2. Yet, under physiological or clinical contexts, the precise antimicrobial mechanisms of copper-based materials remain incompletely understood and warrant further investigation.

### 3.1 Antibacterial mechanisms mediated by conventional copper and copper alloys

Copper and its alloys exhibit broad-spectrum antimicrobial activity through both contact-mediated killing and copper ion release, involving Cu^2+^ and Cu^+^ species (Vincent et al., 2018). While the precise molecular mechanisms of contact killing remain partially understood, it is well established that copper ions accumulate on microbial membranes, dissipate transmembrane potentials, and trigger membrane depolarization. This disruption compromises membrane integrity, resulting in rupture, cytoplasmic leakage, and ultimately cell death (Yu et al., 2024). Notably, SARS-CoV-2 has shown pronounced susceptibility to copper surfaces (Van Doremalen et al., 2021). In addition, Cu^2+^ ions have been reported to inhibit papain-like protease two and degrade viral RNA (Rani et al., 2021).

In aqueous and aerobic environments, copper gradually releases ions that initially bind to thiol groups in glycoproteins on microbial surfaces (Długosz et al., 2025). These ions subsequently interact with membrane phospholipids and proteins, enhancing permeability and triggering localized membrane disruption and cytoplasmic leakage (Ren et al., 2025). Upon internalization, copper ions bind intracellular biomolecules—including proteins and nucleic acids—leading to enzyme inactivation, disruption of electron transport, and interference with essential metabolic pathways (Macomber and Imlay, 2009). Copper can also directly engage with DNA and RNA, inducing strand breaks, structural distortions, mutations, or degradation, thereby hindering microbial proliferation and viral replication. Moreover, copper targets iron–sulfur cluster assembly proteins, such as IscU and IscA. Under anaerobic conditions, intracellular copper accumulation in *E. coli* disrupts Fe–S cluster biogenesis, ultimately compromising bacterial growth and viability (Tan et al., 2017).

Copper-induced oxidative stress significantly contributes to its antimicrobial efficacy. Through Fenton-like reactions and ionic interactions, copper catalyzes the generation of ROS, including superoxide anions (O_2_^−^), hydroxyl radicals (•OH), and hydrogen peroxide (H_2_O_2_) (Warnes et al., 2012; Li W. et al., 2018). These ROS initiate lipid peroxidation, enzyme inactivation, and nucleic acid damage—cascading events that ultimately compromise cellular viability. Remarkably, copper exhibits potent toxicity even under anaerobic conditions, where ROS generation is minimal. This ROS-independent lethality is attributed to mismetallation, wherein copper displaces essential metal cofactors such as Fe^2+^ and Zn^2+^ in metalloproteins, resulting in functional inactivation (Zuily et al., 2022). In anaerobic *E. coli*, copper exposure leads to protein aggregation, likely mediated by Cu-thiol and Cu-histidine interactions in cysteine- and histidine-rich proteins. Strains deficient in molecular chaperones such as DnaK or trigger factor exhibit heightened copper sensitivity, underscoring the role of chaperone-assisted folding in mitigating proteotoxic stress (Zuily et al., 2022). Fu et al. recently introduced a hypoxia-enhanced copper ion interference strategy employing photodynamically activated copper coordination polymer microneedles. This system creates a localized hypoxic microenvironment, promoting sustained Cu^+^ release while concurrently downregulating multicopper oxidase activity, thereby amplifying bactericidal efficacy (Fu et al., 2025). Despite these advancements, the molecular mechanisms underlying copper-induced lethality remain incompletely understood. Excess intracellular copper disrupts core metabolic processes by binding to fatty acylated intermediates in the tricarboxylic acid (TCA) cycle, destabilizing Fe-S cluster-containing proteins, and inducing metabolic collapse—together culminating in irreversible cellular dysfunction and death (Tsvetkov et al., 2022).

Cuproptosis-like death in bacteria describes a copper-induced cell death mechanism, analogous to eukaryotic cuproptosis, but specific to bacterial cells. In bacteria, Cu^+^/Cu^2+^ ions disrupt TCA cycle enzymes and Fe-S cluster biogenesis, inducing similar metabolic failure but lacking the protein aggregation hallmark of eukaryotic systems (Tan et al., 2017; Hua et al., 2024). This process involves the disruption of the TCA cycle and associated metabolic pathways by copper ions, culminating in oxidative stress and eventual cellular demise (Hua et al., 2024). To potentiate this lethal mechanism, a novel MnO_2_-loaded copper metal–organic framework (MCM) was engineered to reprogram bacterial respiration and enhance cuproptosis-like death. In hypoxic biofilms, MCM catalyzes H_2_O_2_ decomposition and *in situ* oxygen generation, alleviating biofilm-associated hypoxia and shifting bacterial metabolism from anaerobic glycolysis toward aerobic respiration—thereby increasing TCA cycle activity and susceptibility to copper-induced toxicity (Luo et al., 2024). Copper ions specifically target iron–sulfur cluster proteins (e.g., IscU, IscA) and TCA cycle enzymes, particularly dihydrolipoamide S-acetyltransferase (DLAT), causing intracellular copper accumulation, DLAT aggregation, and triggering a cascade of metabolic collapse (Tan et al., 2017; Tsvetkov et al., 2022). Simultaneously, hypoxia reversal reactivates immune cell function and promotes osteogenesis and angiogenesis, while oxygen-rich environments enhance macrophage activity, supporting bacterial clearance (Luo et al., 2024). This spatiotemporal modulation of the microenvironment presents a promising strategy for biofilm eradication and concurrent tissue regeneration. The efficacy of this approach has been demonstrated across multiple studies: Luo et al. showed *S. aureus* biofilm eradication *in vivo* via this mechanism (Luo et al., 2024), while Hua et al. demonstrated similar effects in *P. aeruginosa pneumonia* models, with aerobic respiration amplifying cuproptosis (Hua et al., 2024). However, while metabolic reprogramming via MnO_2_-loaded copper frameworks has proven effective in *S. aureus* and *P. aeruginosa*, validation in diverse strains (e.g., *Klebsiella pneumoniae*, *Acinetobacter baumannii*) and multispecies biofilms is needed to confirm universality (Kuyukina et al., 2025). Additionally, *in vitro* models may not fully capture physiological complexity, necessitating further *in vivo* studies (Luo et al., 2024).

Copper demonstrates potent virucidal activity against a broad spectrum of both enveloped and non-enveloped viruses, including single- and double-stranded RNA and DNA viruses. The effect is particularly pronounced for enveloped viruses such as SARS-CoV-2 and influenza virus (Warnes et al., 2012), but also extends to resilient non-enveloped viruses like norovirus and rotavirus (Albalawi et al., 2024). Copper impairs viral infectivity through multiple mechanisms. By disrupting the lipid bilayer of viral envelopes or capsids, copper causes structural disintegration and subsequent inactivation (Mertens et al., 2022; Manuel et al., 2015). It can also bind to viral surface proteins—such as the spike glycoprotein (S protein) of SARS-CoV-2—altering their conformation and thereby hindering host receptor engagement, ultimately preventing viral entry and replication (Hilton et al., 2024). As a transition metal with oligodynamic properties, copper is capable of displacing essential metal ions in metalloproteins, leading to enzymatic inactivation in viral or microbial systems. Furthermore, copper directly interacts with viral nucleic acids, inducing irreversible degradation. Given their limited capacity for nucleic acid repair, viruses are particularly susceptible to copper-mediated genomic damage (Rakowska et al., 2021). Beyond ionic mechanisms, copper surfaces exert direct antiviral effects: upon physical contact, viral particles undergo rapid structural breakdown independent of ion diffusion, highlighting the critical role of surface-mediated inactivation in the overall antiviral efficacy of copper-based materials (Hilton et al., 2024).

### 3.2 Antimicrobial mechanisms mediated by copper-based nanomaterials

Copper-based nanomaterials, including copper nanoparticles (Cu NPs), Cu_2_O, and CuO, offer enhanced antimicrobial efficacy compared to bulk copper due to their high surface-area-to-volume ratio and rapid ion release (Woźniak-Budych et al., 2023). These materials disrupt microbial membranes, induce ROS, and interfere with metabolic processes, with mechanisms varying by nanomaterial type and environmental conditions. Copper nanomaterials exhibit multifaceted antimicrobial action, summarized in Table 1. Membrane disruption occurs via ion-mediated depolarization and physical contact, while ROS generation causes oxidative damage. The “Trojan horse” mechanism involves nanoparticle internalization, releasing copper ions intracellularly to amplify toxicity. Biofilm inhibition is enhanced by ROS and quorum sensing (QS) disruption, though efficacy depends on concentration and exposure duration (Mammari et al., 2022). In addition to their antibacterial effects, copper nanoparticles have demonstrated antiviral potential by directly interacting with viral envelope proteins or host cell receptors, thereby obstructing viral entry and subsequent replication (Bhatti and DeLong, 2023).

**TABLE 1:** Comparative mechanisms of conventional copper and copper-based nano-materials.

Mechanism	Conventional copper	Copper-based nano-materials
Ion Release	Gradual Cu^2+^/Cu^+^ release; limited by oxide layers	Rapid Cu^2+^/Cu^+^ release; due to high surface area
ROS Generation	Moderate; via Fenton-like reactions	Enhanced; high redox activity
Membrane Disruption	Slower; contact-mediated	Rapid; nanotextured surface contact-killing
Biofilm Inhibition	Limited; depends on surface roughness	Strong; disrupts QS and EPS
Trojan Horse Mechanism	Not observed	Internalized; amplifying ROS production
Cuproptosis-Like Death	Not observed	Enhanced; Disrupts TCA cycle, enhances lethality

Compared to bulk copper, nanocopper exhibits markedly enhanced microbicidal efficacy, largely attributable to its elevated surface-area-to-volume ratio, accelerated ion release kinetics, and efficient Fenton-like redox activity (Zhao et al., 2023). Through redox cycling, nanocopper facilitates the generation of ROS, including hydroxyl radicals, superoxide anions, and hydrogen peroxide. These oxidative intermediates elicit widespread cellular damage by inducing lipid peroxidation, protein denaturation, and nucleic acid fragmentation via both enzymatic and non-enzymatic pathways (Salah et al., 2021; Warnes et al., 2012; Li W. et al., 2018).

Additionally, nanocopper undergoes rapid dissolution, releasing Cu^+^ and Cu^2+^ ions that interact with membrane phospholipids and intracellular targets, thereby amplifying oxidative stress (Wei et al., 2024). Importantly, extracellular ion release alone does not fully account for the observed antimicrobial potency. Rather, the internalization of copper nanoparticles and their subsequent intracellular degradation lead to a localized surge of bioactive copper ions within the cytoplasm. This intracellular ion burst triggers excessive ROS generation, ultimately causing widespread cellular dysfunction and death—a phenomenon commonly described as the “Trojan horse” mechanism of copper (Ma et al., 2022).

Bacterial biofilms, composed of self-secreted extracellular polymeric substances (EPS), provide enhanced protection against environmental stressors and antimicrobial agents. These complex, multicellular structures—often found on moist surfaces—pose a formidable challenge to treatment. As biofilms mature, their resistance to copper-based antimicrobials significantly increases. Among various disruption strategies, the generation of ROS at the nanoparticle interface plays a central role in microbial inactivation and biofilm dispersal (Sedighi et al., 2024). For instance, chloride- and nitrite-enhanced Cu-Fenton chemistry has demonstrated effective biofilm degradation through accelerated ROS production (Wang et al., 2017b; Wang et al., 2021).

Copper nanoparticles further inhibit biofilm formation by disrupting QS, the microbial communication system governing biofilm development (Desai et al., 2021). Copper (II) complexes coordinated with aromatic nitrogen-containing heterocycles have emerged as potent QS inhibitors, particularly in *Pseudomonas aeruginosa* (Glišić et al., 2016). The expanding application of copper-based nanomaterials in oral hygiene and wound care has garnered attention. Notably, copper-based carbon dots (Cu-CDs) have demonstrated the ability to inhibit *Streptococcus mutans* adhesion and promote biofilm dispersion, positioning them as next-generation antibiofilm agents for clinical use (Liu et al., 2022).

To further enhance antibiofilm efficacy, copper-based nanotherapeutics inducing cuproptosis-like bacterial death have been developed. Lung-targeting Cu_2_O–BSO nanoparticles penetrate mucus barriers and amplify cuproptosis by depleting glutathione via buthionine sulfoximine (BSO), simultaneously disrupting QS, biofilm formation, and bacterial virulence while promoting macrophage-mediated clearance (Hu et al., 2025). In parallel, PEG4000-assisted CuCo_2_O_4_ nanoflowers exhibit enhanced multienzymatic activities, generating ROS and depleting GSH to disrupt bacterial metabolism. Cu^2+^ overload compromises the TCA cycle and respiration, ultimately triggering cuproptosis-like death. Both platforms demonstrate robust *in vivo* efficacy against MRSA pneumonia and biofilm-infected wounds, offering a synergistic strategy for combating drug-resistant pathogens (Wang et al., 2024).

Copper-based nanomaterials typically require high concentrations to effectively inhibit biofilm formation (Siddique et al., 2024). However, elevated copper levels raise concerns regarding environmental toxicity (Wang and Wang, 2022). Compounding this issue, emerging evidence suggests that copper may accelerate the dissemination of AMR (Zhang et al., 2019), emphasizing the need to elucidate the mechanisms underlying copper-mediated biofilm disruption and to define a safe yet efficacious therapeutic window. In a pivotal study, Kuyukina et al. systematically examined the dual effects of Cu^2+^ released from copper oxide nanoparticles (CuO NPs) on bacterial biofilms and host cell resilience, providing key insights for the rational design of next-generation anti-biofilm nanomaterials (Kuyukina et al., 2025). At sublethal concentrations, CuO NPs exhibit limited affinity for bacterial cell walls, inducing only minor structural perturbations. Interestingly, the resulting increase in surface roughness enhances cellular adhesion, paradoxically promoting biofilm formation. In *Rhodococcus* spp., intracellular ROS levels initially rise but later decline, suggesting an adaptive oxidative stress response to prolonged low-dose CuO NP exposure. This is accompanied by moderate accumulation of proteins and polysaccharides in the extracellular matrix, supporting a gradual increase in biofilm biomass. Conversely, at higher concentrations, CuO NPs aggregate extensively on bacterial surfaces, inhibiting adhesion and co-aggregation—the critical early steps of biofilm formation. Some nanoparticles penetrate the cell envelope and accumulate intracellularly, triggering a burst of ROS production. This culminates in membrane rupture, metabolic collapse, and widespread bacterial death in an avalanche-like cascade. The sharp elevation in ROS and concurrent suppression of metabolic activity within *Rhodococcus* biofilms indicate a failure of most cells to mount a protective oxidative stress response. A minority of surviving cells may activate DNA repair pathways, upregulate antioxidant enzymes, or increase EPS production to mitigate nanoparticle-induced damage. Biofilms formed under high CuO NP stress exhibit significantly increased lipid content (∼27%) and a twofold enrichment of proteins and polysaccharides, likely resulting from matrix debris of lysed cells and reduced viable biomass. Notably, intracellular carotenoid levels are markedly elevated, potentially functioning as antioxidant shields against ROS-mediated cytotoxicity. While CuO NP exposure does not visibly alter cellular morphology within biofilms, it induces plasma membrane damage and cytoplasmic heterogeneity, possibly due to dysregulated ion fluxes (Na^+^, Ca^2+^, Mg^2+^ and K^+^) and perturbed metabolic stress responses (Kuyukina et al., 2025). However, it is important to note that these findings are derived from *in vitro* models using a single bacterial genus *Rhodococcus*, and may not fully capture the complexity of multispecies biofilms or host-associated environments. Further studies are needed to validate these mechanisms across broader microbial communities and under physiologically relevant conditions.

A defining characteristic of copper nanoparticles is their exceptionally high specific surface area, a property that underpins their potent antimicrobial activity. This enlarged interfacial domain enables intimate contact with microbial membranes, facilitating a spectrum of direct physicochemical interactions. Notably, localized mechanical rupture, membrane perforation, and pressure-induced deformation collectively undermine the structural integrity of microbial cells. This phenomenon—termed the “nanotextured surface contact-killing effect”—has gained increasing recognition as a pivotal mechanism underlying nanoparticle-driven antimicrobial efficacy (Bhatti and DeLong, 2023). While increasingly recognized, the precise contribution of this contact-mediated disruption under physiological conditions, and its potential cytotoxicity to host tissues, remains incompletely defined and warrants further systematic investigation.

## 4 Copper and copper-based materials: fabrication strategies and antimicrobial properties

**FIGURE 3:**
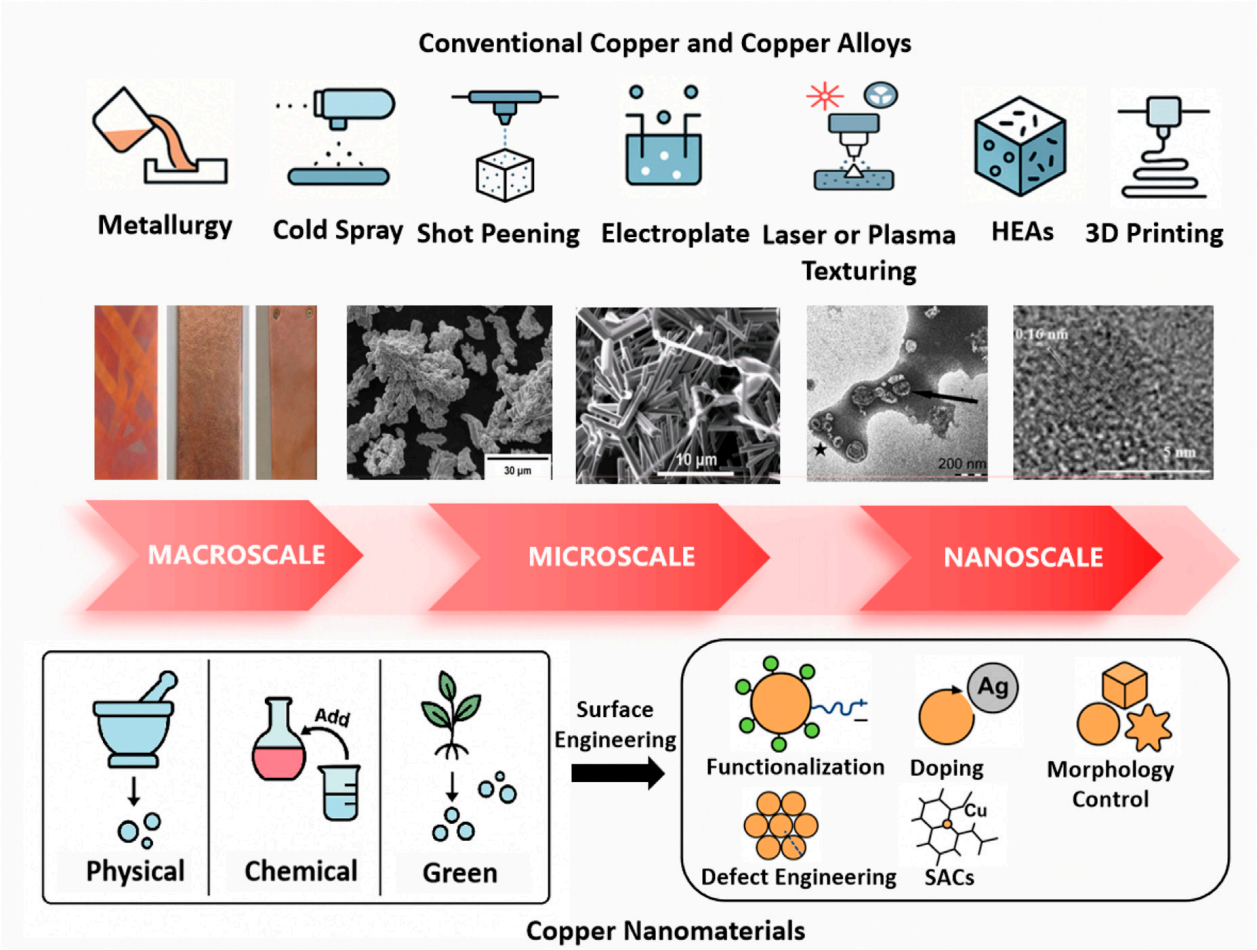
Schematic representation of common copper and copper-based materials preparation techniques.

Copper-based materials exhibit broad-spectrum antimicrobial activity via multiple interrelated mechanisms, including controlled ion release, disruption of membrane integrity, and the induction of oxidative stress. Traditional forms, such as pure copper, brass, and bronze, have been extensively utilized in high-touch surfaces and infrastructure, where their efficacy is modulated by alloying elements, grain structure, and surface finishing techniques. In contrast, copper-based nanomaterials—including nanoparticles, nanowires, and composite platforms—demonstrate superior microbicidal performance at significantly lower concentrations, attributed to their increased surface reactivity and enhanced generation of reactive oxygen species. Owing to their nanoscale physicochemical properties, these materials can be seamlessly integrated into functional coatings, biomedical devices, and antimicrobial textiles, enabling localized and rapid microbial inactivation. The schematic overview of their preparation techniques is shown in Figure 3.

### 4.1 Conventional copper and copper alloys: microstructure optimization and surface engineering toward enhanced antimicrobial performance

Conventional copper and its alloys, including brass and bronze, have long been recognized for their inherent antimicrobial properties, and are extensively employed in plumbing systems, kitchenware, high-touch surfaces such as door handles, and medical instruments. Notably, several copper alloys have been officially approved by the U.S. Environmental Protection Agency (EPA) for antimicrobial use within healthcare infrastructure (Borkow and Gabbay, 2009; Dauvergne and Mullié, 2021). The fabrication of copper-based materials typically involves traditional methods such as melting and casting, powder metallurgy, and electroplating, although emerging techniques continue to expand the manufacturing landscape (Rodrigues et al., 2021). The antimicrobial efficacy of these materials is modulated by multiple physicochemical factors, including copper content, surface roughness, microstructural architecture, environmental parameters, alloying constituents, and the composition and thickness of the surface oxide layers (Georgakopoulos-Soares et al., 2023; Birkett et al., 2022).

In general, higher copper content, increased surface roughness, refined microstructures, humid or acidic environments, the incorporation of reactive alloying elements, and thinner oxide layers are all positively correlated with enhanced antimicrobial activity (Birkett et al., 2022; Zhang et al., 2021; Ding et al., 2019). The antimicrobial efficacy of copper can be substantially improved through surface engineering strategies such as cold spraying and shot peening. Cold spray treatment induces localized grain refinement and dynamic recrystallization, resulting in high-density grain boundaries and submicron structures that accelerate copper ion release and strengthen antibacterial performance (Sousa et al., 2021; Razavipour et al., 2022). Bulk surface nanocrystallization, a more advanced approach, further increases dislocation density and refines grain size, facilitating faster ion diffusion and yielding superior antimicrobial outcomes (Acharya et al., 2021; Azadmanjiri et al., 2015). To elucidate the relationship between crystallographic orientation and antimicrobial activity, Hirota et al. investigated copper thin films exhibiting distinct crystal orientations. Their study revealed that Cu(100)-oriented films, which mimic single-crystal copper, form a stratified Cu/Cu_2_O/CuO architecture from the substrate surface outward. This layered structure accelerates Cu_2_O formation and introduces surface defects such as steps, kinks, and facets—structural features that enhance ROS generation and improve antimicrobial efficacy against *E. coli*, *S. aureus*, and A/Hong Kong influenza virus (Hirota et al., 2025).

Upon prolonged exposure to ambient air and moisture, copper surfaces undergo sequential oxidation, initially forming a reddish-brown Cu_2_O layer followed by a black CuO layer, accompanied by a gradual decline in antimicrobial efficacy. The physicochemical properties of these oxides play a central role in modulating corrosion resistance, redox behavior, and biocidal performance. Cu_2_O, which releases monovalent Cu^+^ ions, demonstrates superior redox activity and potent antimicrobial efficacy compared to CuO, rendering it more suitable for rapid-disinfection applications such as medical coatings, functional textiles, and air filtration systems. In contrast, CuO exhibits greater chemical stability but reduced antimicrobial activity, making it preferable for long-term antimicrobial surfaces and catalytic applications (Park et al., 2024; Sunada et al., 2012). For instance, Sunada et al. reported that solid-state cuprous compounds—including Cu_2_O, Cu_2_S, CuI, and CuCl—exhibit significantly greater antiviral activity than either silver or their cupric analogues. Notably, Cu_2_O was more effective than CuO in protein adsorption and denaturation, which underpins its enhanced bioactivity (Sunada et al., 2012). In parallel, Minoshima et al. demonstrated that Cu_2_O inactivates influenza A virus and bacteriophage Qβ by denaturing viral surface proteins, whereas CuO exhibited minimal antiviral activity (Minoshima et al., 2016).

Despite copper’s well-documented antimicrobial properties, real-world applications—such as ship hulls, water pipelines, and aquaculture systems—remain vulnerable to biofilm formation. This limitation often stems from insufficient copper ion release under complex environmental conditions, undermining the primary biocidal mechanism. Biofilm development involves initial microbial adhesion, colonization, and maturation into structured communities that can shield pathogens from copper-mediated killing (Shineh et al., 2023). During early-stage colonization, smoother surfaces show reduced microbial attachment, whereas in later stages, surfaces modified via shot peening exhibit enhanced antibiofilm performance compared to untreated or cold-sprayed copper, likely due to more efficient ion diffusion and localized ion accumulation. While increased surface roughness can promote ion release and improve antimicrobial efficacy, it also accelerates corrosion and may compromise long-term material durability (Razavipour et al., 2022). Moreover, excessive ion release raises biosafety concerns in certain settings. To address these challenges, multifaceted strategies have emerged. Surface nanostructuring amplifies contact-killing through enhanced reactivity and mechanical disruption (Acharya et al., 2021; Azadmanjiri et al., 2015), while alloying with elements such as Ag, Zn, or Ni introduces synergistic antimicrobial effects (Wang et al., 2016; Parimaladevi et al., 2018). Functional coatings—including Cu-based nanocomposites and polymeric modifiers—prolong efficacy and improve biocompatibility (Butot et al., 2021; Butler et al., 2023). Advanced surface engineering approaches, such as laser texturing and plasma activation, further refine oxide composition and surface energy, optimizing both antimicrobial performance and environmental stability (Walkowicz et al., 2025).

Concurrently, advancements in materials processing technologies have facilitated the precise engineering of copper-based surfaces, enabling sustained antimicrobial performance with reduced environmental footprint. High-entropy alloys (HEAs) and high-entropy coatings (HECs)—comprising five or more principal elements in near-equiatomic ratios—have emerged as promising candidates for antimicrobial applications when doped with copper. Their highly disordered atomic configurations promote homogeneous copper ion release, thereby offering tunable biocidal efficacy with minimal ecological burden (Li Z. et al., 2021). Yu et al. engineered AlCoCrFeNiCu_0.5_ HEAs with uniformly dispersed Cu nanophases, mitigating phase segregation and brittleness while enhancing toughness, corrosion resistance, and antifouling performance (Yu et al., 2021). Similarly, Kuptsov et al. fabricated FeCrNiCo-(Cu) HECs using vacuum electro-spark deposition, resulting in uniform microstructures with Cu stabilized in solid solution. These coatings demonstrated excellent corrosion resistance, rapid self-passivation, and robust antibacterial activity against *Bacillus cereus*, underscoring their potential for deployment in aggressive marine environments (Kuptsov et al., 2023).

Additive manufacturing (AM), commonly referred to as 3D printing, has further expanded the design space for copper-based materials by enabling the fabrication of geometrically complex, porous architectures with optimized surface area and material utilization. Techniques such as selective laser melting (SLM) have yielded microporous Cu-W-Ag structures with enhanced mass transport and high surface reactivity. Notably, John et al. demonstrated that such 3D-printed architectures exhibit potent antiviral activity against SARS-CoV-2, highlighting the integration of alloy composition, topological control, and advanced processing techniques as a powerful strategy for developing next-generation antimicrobial surfaces (Robinson et al., 2021).

### 4.2 Copper nanomaterials: nanotechnology and advanced surface engineering for next-generation antimicrobial strategies

Nanostructured copper exhibits superior antimicrobial performance compared to its bulk counterparts, owing to its rapid ion release kinetics, elevated surface reactivity, and tunable physicochemical properties that are crucial for microbial membrane disruption and biofilm penetration (Ghezzi et al., 2022). In contrast to traditional copper alloys, nano-copper materials allow precise control over particle size, morphology, and composite integration, thereby significantly enhancing their biocidal efficacy (Molahalli et al., 2024). These advancements mark a paradigm shift into the “nano-copper era,” wherein nanoscale engineering enables unprecedented stability, biocompatibility, and broad-spectrum antimicrobial activity. Copper nanoparticles (CuNPs) have demonstrated potent antimicrobial efficacy against a wide array of pathogens, including viruses such as HIV, SARS-CoV-2, HBV, HCV, HSV, and RSV, as well as bacterial species like *E. coli*, *S. aureus*, *Salmonella* spp., and *Streptococcus* spp. (Solangi et al., 2024). Their enhanced bioactivity relative to bulk copper is primarily attributed to the higher density of grain boundaries at the nanoscale, which facilitates accelerated copper ion release and promotes the generation of ROS (Sundberg et al., 2015).

A variety of physical, chemical, and biological methods have been developed for the synthesis of copper-based nanoparticles (Crisan et al., 2021). Among these, chemical approaches, including sonochemical reduction, hydrothermal synthesis, electrochemical deposition, and chemical reduction, are the most widely adopted due to their versatility, scalability, and control over particle characteristics (Pricop et al., 2025). Pricop et al. synthesized Cu NPs via chemical reduction method, exhibited high stability, tunable size, and strong antimicrobial activity against both Gram-positive and Gram-negative bacteria (Pricop et al., 2025). Physically synthesized nanoparticles, produced by techniques such as simple ball milling, physical vapor deposition, or laser ablation, offer uniform distribution and solvent-free purity, though their application is limited by high energy demands and equipment costs (Pricop et al., 2025; Wei et al., 2020). Wei et al. synthesized CuO-biochar via simple ball milling, exhibiting strong adsorption capacity and potential for water purification (Wei et al., 2020). Hesabizadeh et al. synthesized CuO/Cu_2_O NPs via pulsed laser ablation, demonstrating rapid cell wall disruption and broad-spectrum antibacterial efficacy against major foodborne pathogens at a low concentration of 3 ppm within 5 h (Hesabizadeh et al., 2023). In contrast, biological or green synthesis, utilizing plant extracts, bacteria, or fungi as reducing and stabilizing agents, offers a sustainable, cost-effective, and biocompatible route for nanoparticle production and has emerged as a promising strategy for large-scale, low-toxicity manufacturing (Chaerun et al., 2022). Nkosi et al. biosynthesized Cu NPs using a carbohydrate-based bioflocculant from *Proteus mirabilis*, showing potent antibacterial activity (Nkosi et al., 2025). Javid-Naderi et al. biosynthesized CuO NPs with okra extract, and further doped with silver, exhibited enhanced antimicrobial activity (Javid-Naderi et al., 2025).

Regardless of the synthesis route, copper-based nanoparticles (CuNPs) inherently exhibit robust antimicrobial properties. However, to further enhance their functional performance, stability, and specificity, surface engineering strategies are increasingly indispensable. Several advanced modification techniques have emerged as particularly effective: (1) Surface Modification and Functionalization: Tailoring the surface topography and charge—such as introducing positively charged functional groups to promote electrostatic interaction with negatively charged microbial membranes, or conjugating biomolecules to enable targeted microbial recognition—can significantly improve microbial adhesion, inactivation efficiency, and selectivity while minimizing off-target interactions. Woźniak-Budych et al. reported that cellulose acetate membranes embedded *in situ* with copper(I) oxide nanoparticles, stabilized by polyvinylpyrrolidone and sulfobetaine to limit copper ion leakage, exhibited markedly enhanced antibacterial activity against *S. aureus* and superior antifouling properties under physiological conditions, highlighting their potential in next-generation hemodialysis systems (Woźniak-Budych et al., 2024). Glutamic acid-coated copper oxide nanoparticles (GA-CuO NPs), covalently functionalized onto medical-grade silicone tubing via an oxysilane linker, demonstrated broad-spectrum efficacy, including activity against MDRpathogens (Hall et al., 2024). (2) Doping Strategies: Incorporating secondary metals such as silver or zinc into CuNPs produces nanocomposites with synergistic antimicrobial effects. Ag-doped copper nanoparticles (Cu–Ag NPs) have shown a >100-fold increase in antiviral activity against SARS-CoV-2, attributed to a sacrificial anode mechanism whereby silver accelerates copper ion release (Patlejchová et al., 2023). Similarly, co-doping CuO nanocomposites with silver and magnesium (optimal Cu:Ag:Mg ratio of 94:3:3) improved both antimicrobial potency and cytocompatibility (Kasi et al., 2024). (3) Morphology Control: Engineering specific nanostructures—such as nanowires, nanosheets, and other high-aspect-ratio forms—increases surface area and exposure of active sites, thereby enhancing antimicrobial performance. Park et al. synthesized Cu_2_O nanoparticles in spherical, octahedral, and cubic shapes via chemical reduction and found that cubic Cu_2_O retained the highest antimicrobial activity under prolonged thermal and humid stress, owing to its superior oxidation resistance (Park et al., 2024). (4) Defect Engineering: Introducing lattice defects into copper nanocrystals enhances their redox activity and surface reactivity. Lasemi et al. used femtosecond laser ablation to generate crystalline Cu_0.70_Zn_0.3_0 alloy nanoparticles with abundant structural defects and periodic surface features. The resulting low-coordinated surface atoms exhibited elevated catalytic and antimicrobial activity, illustrating the synergy between crystallographic imperfections and biological functionality (Lasemi et al., 2024). (5) Single-Atom Catalysis (SACs): SACs represent a state-of-the-art strategy in which isolated copper atoms are stabilized on solid supports, offering unique electronic properties and maximized atom utilization. In antimicrobial contexts, SACs enable strong biological effects at ultralow metal concentrations, minimizing cytotoxicity while maximizing therapeutic efficacy. Zhao et al. reported that copper single atoms anchored on nitrogen-doped mesoporous carbon nanospheres efficiently generated superoxide radicals under ambient conditions, leading to broad-spectrum antibacterial activity and accelerated wound healing *in vivo* (Zhao et al., 2021). Lin et al. further demonstrated that SACs based on Cu anchored on graphitic carbon nitride (SA-Cu/g-C_3_N_4_) efficiently activated hydrogen peroxide in a photo-Fenton-like process, achieving complete inactivation of MRSA and CRAB within 5 min, and eliminating ESBL-producing *E. coli* and vancomycin-resistant *Enterococcus* (VRE) within 10 and 30 min, respectively (Lin et al., 2021).

Collectively, the integration of copper nanotechnology with advanced surface engineering has significantly expanded the toolkit for antimicrobial material development. These engineered nanostructures provide promising avenues to overcome key limitations of conventional copper-based systems. Moreover, they exhibit substantial potential in addressing antimicrobial resistance, preventing nosocomial infections, and countering emerging viral threats.

## 5 Antimicrobial frontiers: translational applications of copper and copper-based materials

**FIGURE 4:**
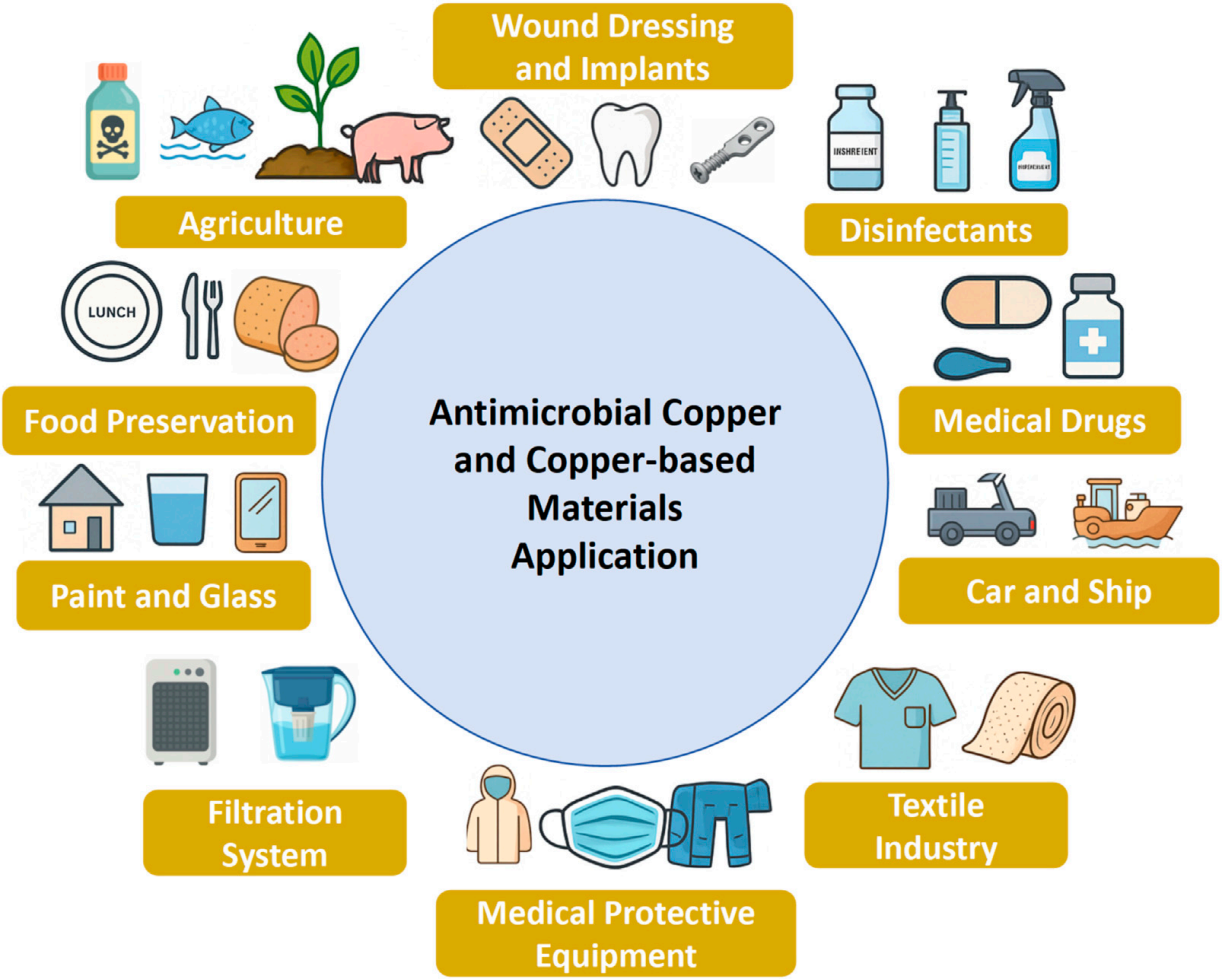
Antibacterial applications of copper and copper-based materials.

Amid escalating global health challenges and the alarming surge in antibiotic-resistant bacteria and emerging viral pathogens, copper and its derivatives have gained renewed attention due to their potent and broad-spectrum antimicrobial and antiviral properties (Bisht et al., 2022). These materials are now extensively deployed across healthcare, agriculture, animal husbandry, water treatment, and the textile industry as illustrated in Figure 4. Copper ions, particularly those derived from salts and coordination complexes, display strong biocidal activity and are employed in various roles as disinfectants, algicides, fungicides, nematicides, and antifouling agents (Pourmadadi et al., 2024). Furthermore, copper-based compounds hold considerable promise as antiviral therapeutics (Devaraji et al., 2024). While conventional copper and its alloys offer robust and chemically stable antimicrobial activity, their widespread application is constrained by cost and scalability. Advances in nanotechnology have enabled the fabrication of copper nanocoatings that not only minimize material usage but also amplify antimicrobial efficacy. These nanoscale formulations exhibit potent, broad-spectrum antimicrobial properties, with tunable surface chemistry to meet diverse functional demands. Critically, next-generation antimicrobial surfaces must combine pathogen eradication, biofilm inhibition, biocompatibility, and environmental sustainability for durable and safe application (Ana et al., 2025; Pontin et al., 2021). Comparative data on various copper-based materials and their respective application domains are summarized in Tables 2, 3.

**TABLE 2:** Preparation methods, antimicrobial characteristic, and applications of conventional copper and copper-based materials.

Material types	Preparation methods	Characteristic	Application	References
Copper and alloys	Conventional metallurgy: solid pure copper, hot coated copper, film copper	All copper exhibited strong antibacterial activity against *S. aureus* and *P. aeruginosa*, >90% inhibition of HCoV-229E, and durability exceeding 200 washing cycles	Antimicrobial surfaces	Charles et al. (2024)
	Conventional metallurgy: pure copper, copper alloy C22000 (90% Cu, 10% Zn) and C21000 (95% Cu, 5% Zn)	Antiviral activity against SARS–CoV–2: Copper alloy C21000 > copper alloy C22000 > pure copper	Antimicrobial surfaces	Nakano et al. (2025)
	Conventional metallurgy: Cu15Zn, Cu18Ni20Zn and pure copper	All copper-based surfaces fully inactivated SARS-CoV-2 within 10 min, with pure copper showing the highest potency	Antimicrobial surfaces	Lorenzetti et al. (2024)
	Conventional metallurgy: Ti–Cu alloy	Sustained antibacterial activity emerged only at copper contents above 5%	Medical implants and devices	Liu et al. (2014)
	Conventional metallurgy: Ti6Al4V-Cu alloy	Effectively inhibits MRSA and biofilm formation while maintaining high biocompatibility	Medical implants and devices	Zhuang et al. (2021)
	3D-printing: Ti6Al4V/Cu	Ti6Al4V/Cu could inhibit bone resorption caused by microbial infection. Increasing the Cu content in material to 9.7 wt% achieves 99% bacterial reduction	Medical implants and devices	Nikolaeva et al. (2023)
	HEAs	Cu-containing HEAs achieve >99.99% influenza virus H1N1 and enterovirus 71 (EV71). inactivation within 24 h	Industrial manufacturing: automotive, and maritime	Li et al. (2021a)
Micrometer copper particles	Physical grinding; microparticles of Cu_2_O, CuO, Cu_2_S, and CuS	Cu_2_O and Cu_2_S microparticles (0.5–100 μm) showed superior antiviral activity (5-log reduction) over CuO and CuS (3-log); CuS-infused nylon masks inhibited SARS-CoV-2 by up to 80%	Antimicrobial surfaces	(Sunada et al., 2012), (Hewawaduge et al., 2021)
Micrometer copper film	Single-crystal copper growth + mechanical polishing + electrochemical polishing + ultra-high vacuum (UHV) cleaning: Cu(100) surface	The 18-µm-thick Cu(100) surface exhibited potent antimicrobial and antiviral activity, achieving >4-log reductions in both bacterial and viral loads	Industrial manufacturing: automotive, and maritime	Hirota et al. (2025)
	Electrostatic sprayin: Cu–Ag composite coatings	The Cu–Ag coating (40 μm) exhibited rapid and efficient virucidal activity against SARS-CoV-2	Antimicrobial surfaces	Mosselhy et al. (2021)
	Electroplating:anodic aluminium oxide (AAO)-copper coatings AAO-Cu	The 2 μm-thick coating inhibited over 95% of *E. coli* growth	Antimicrobial surfaces	Chen et al. (2023)
Copper ions and their complexes	Copper salts: Cu^+^-based formulations	Cu^+^ ions enabled highly transparent coatings with >99.9% inhibition of *S. aureus*	Architectural coating	Culpepper et al. (2025)
	Copper salts: Cu^2+^-based formulations	Cu^2+^ effectively inhibits FCV, DENV, and H9N2, disrupting viral integrity and inducing morphological damage	Antimicrobial surfaces	(Li et al., 2020), (Horie et al., 2008)
	Layer-by-layer self-assembly technology: embedding copper ions into polyelectrolyte multilayer films	Copper-infused fibers rapidly inactivate MHV-3, *E. coli*, and *S. aureus*, demonstrating broad-spectrum antimicrobial efficacy	Textile industry	Calais et al. (2024)
	Drop-drying technique: *in situ* self-assembly of phosphine-ligated copper iodide into cotton fibers	The engineered cotton fibers exhibit potent broad-spectrum antimicrobial activity, achieving up to 99.9% elimination efficiency against MHV, SARS-CoV, and MRSA.	Textile industry	Chen et al. (2024)
	Copper salts: A mixed copper ion solution (CBMS), commercially known as SKYBE-783	CBMS forms tightly adherent copper nanowrinkles (50–100 nm) with potent, broad-spectrum inhibition against MRSA, H3N2, FCV, and SARS-CoV-2	Antimicrobial surfaces and kitchenware	Nishimura et al. (2024)
	Impregnation method: copper-incorporated cellulose (Cu-TOC)	Cu-TOC enabled the rapid release of copper ions, achieving an inhibition efficiency of approximately 90% against IFV, SARS-CoV-2, and FCV.	Paper industry	Hirose et al. (2023)
	Impregnation method: copper-incorporated cotton fibers (Cu-IT)	Cu-IT displays strong antiviral activity against TMV and influenza A, and broad-spectrum antibacterial efficacy against *E. coli*, *S. typhimurium*, *P. aeruginosa*, and *B. subtilis*	Textile industry and medical protective equipment	Qian et al. (2023)
	Impregnation method: Cu–zeolite coating composed of Cu_2_Cl(OH)_3_	The 2.5 µm thick coating markedly reduces coliform levels on surfaces and in air	Antimicrobial surfaces and textile	Fuentes-Alburquenque et al. (2024)
	Impregnation method: loading copper onto gallium-based nanoparticles, yielding liquid metal copper alloys (LMCu)	LMCu eliminated over 99% of bacterial, fungal, and viral pathogens within 5 min, outperforming pure copper in antimicrobial efficacy	Textile and medical protective equipment	Kwon et al. (2021)
	Coordination-based strategies:schiff base ligands, coumarin, imidazole, rosin-derived abietate, diphenylacetic acid, phthalocyanine sulfate, dithiocarbamate, and curcumin	Copper ion complexes exhibit nuclease-mimetic activity, effectively degrading viral surface proteins and achieving >90% antiviral efficacy against SARS-CoV-2, HBV, IPNV, HIV, H3N2, and H5N2. They also display strong antibacterial activity against *S*. *aureus* and *E. coli*	Medical and public health: antiviral drugs, disinfectants, and architectural coatings	Bhandarkar et al. (2023), Gutiérrez et al. (2021), Correa et al. (2023), Choudhary (2022), Bilal et al. (2025), Trofimova et al. (2015), Styczynski et al. (2015), Mufhandu et al. (2023), Al-Jameel et al. (2024)

**TABLE 3:** Preparation methods, antimicrobial performance, and applications of copper-based nano-materials.

Material types	Preparation methods	Characteristic	Application	References
Copper and copper-based nano-film	Laser direct writing:Cu nanofilms	The copper nanofilm, with an approximate thickness of 10 nm, inactivated IBRV through contact-mediated killing and the efficient release of Cu^+^ ions	Antimicrobial surfaces	Zuo et al. (2023)
	Magnetron sputtering:nano-film DLC:Cu	The nanocoating exhibited antibacterial efficacy exceeding 99%, and demonstrated antiviral activity with a reduction greater than 1.63 log_10_	Antimicrobial surfaces	Bakhet et al. (2024)
	Magnetron sputtering:nano-film Cu, CuO and Cu2O	The antiviral efficacy of the nanofilms followed the order: Cu (>5 log_10_) > Cu_2_O (∼3 log_10_) > CuO (<1 log_10_)	Antimicrobial surfaces	Shigetoh et al. (2023)
	Magnetron sputtering:Ultrathin bimetallic Cu-Ag nanofilms	Cu–Ag nanofilms (<5 nm) leveraged the sacrificial anode effect to enhance Cu^2+^ release, yielding superior antiviral efficacy over monometallic Cu or Ag films	Antimicrobial surfaces	Meister et al. (2022)
	Magnetron sputtering:nano-Cu-Zn coated filters	The coated filters exhibited inhibitory activity against *E. coli*, *S. aureus*, and EV71	Textile industry and medical protective equipment	Zhang et al. (2022b)
	Magnetron sputtering:nano-Cu-coated filters	A 30 nm copper coating achieved 99.99% removal of *S. aureus*, *K. pneumoniae*, *E. coli*, and *P. aeruginosa*	Air and water filtration	Jung et al. (2022)
	Phase inversion + laser induction: nano-Cu-LIG-coated filters	The nano films achieved a 6 log_10_ reduction in bacterial and viral loads	Water filtration	Dixit et al. (2025)
Copper and copper-based nanoparticles	High-energy electron beam synthesis: Cu NPs	Cu NPs (∼100 nm, spherical) displayed potent antiviral activity against H1N1, free from copper oxides	Antiviral drugs	Ha et al. (2022)
	Microwave + aqueous blending + high-temperature sintering: MW-CuO/TEG nanocomposites	The nanocomposite, with an average thickness of 32 nm, achieved a 99.9% reduction in HSV-1 viral load	Antiviral drugs	Hamidzade et al. (2024)
	Wet-chemical synthesis: Cu_2_S NPs	Spherical Cu_2_S NPs (∼5 nm) photochemically cleave HBV core antigen and inhibit viral replication, effectively reducing HBV infectivity	Antiviral drugs	Guo et al. (2021)
	Solution precipitation synthesis: CuI NPs	CuI NPs (100–400 nm) exhibited >7-log_10_ reduction in feline calicivirus (FCV) infectivity	Antiviral drugs	Shionoiri et al. (2012)
	Green synthesis: Cu NPs produced using Ficus carica extract	The nanoparticles (∼40 nm) effectively inactivated H1N1, AdV40, and HSV-II.	Antiviral drugs	Hmed et al. (2025)
	Green synthesis: Cu NPs produced using Fruit Extract of Syzygium alternifolium Walp	The Cu NPs (∼20 nm) exhibited potent virucidal activity against NDV.	Antiviral drugs	Yugandhar et al. (2018)
	Green synthesis:CuO NPs produced using Rubia cordifolia bark extract	Spherical CuO NPs (∼50 nm) exhibited excellent broad-spectrum antibacterial activity against *E. coli*, *P. aeruginosa*, *S. aureus*, and *B. cereus*	antiviral agents and environmental remediation	Vinothkanna et al. (2023)
	Green synthesis:CuO NPs produced using *Morinda citrifolia* leaf extract	Spherical nanoparticles (∼50 nm) exhibited potent inhibitory activity against Gram-positive (*B. subtilis*, *S. aureus*), Gram-negative (*E. coli*) bacteria, and fungi (*A. flavus*, *A. niger*, *P. frequentans*)	Antibacterial agents	Priya et al. (2023)
	Green synthesis:CuO NPs produced using Nyctanthes arbor-tristis Linn Leaf extract	CuO NPs (∼30 nm; rectangular, truncated triangular, and spherical) exhibited strong antibacterial activity against *E. coli* and *S. aureus*	Antibacterial agents	Noorafsha et al. (2022)
	Green synthesis:CuO NPs produced using leaf extracts of Ocimum lamiifolium Hochst. ex Benth and Withana somnifera (L) Dunal	Irregular spherical CuO NPs (∼20 nm) exhibited potent antibacterial activity against *S. aureus*, *E. coli*, and *P. aeruginosa*	Antibacterial agents	Mengesha et al. (2024)
	Green synthesis:CuO NPs produced using Pantoea agglomerans CPHN2	Clustered CuO NPs (∼200 nm) exhibited >5-fold enhanced inhibition against *S. aureus* and *B. subtilis* compared to chemically synthesized counterparts	Antibacterial agents	Rani et al. (2025)
	Green synthesis:CuO NPs produced using *Centella asiatica* leaf extract	Elliptical CuO NPs (∼30 nm) exhibited superior antibacterial activity against *E. coli* and MRSA compared to Ag-NPs, with additional wound healing potential	Antibacterial agents	Agarwal et al. (2025)
	Green synthesis:Cu NPs produced using Terminalia bellirica Fruit Extract	The ultrasmall Cu NPs (2–7 nm) demonstrated strong antibacterial activity	Antibacterial agents	Viswadevarayalu et al. (2016)
	Chemical deposition: Se-NPs/Cu_2_O NPs	Spherical Se-NPs/Cu_2_O NPs (∼100 nm) shown ∼5-fold lower MICs for Gram-positive bacteria, but ∼4-fold higher MBCs, likely due to enhanced adsorption and thicker peptidoglycan barriers	Antibacterial agents	Asaad et al. (2025)
	Flame spray synthesis: amorphous tricalcium phosphate nanoparticles (CuTCP) incorporated into a PLGA matrix to form PLGA/CuTCP bone nanocomposites	CuTCP NPs (∼20 nm, spherical) exhibited potent antibacterial and pro-angiogenic activities	Medical fillers	Näf et al. (2024)
	Green synthesis: ZnO-CuO NPs produced using Pleurotus ostreatus spent mushroom substrate extract	Quasi-spherical ZnO-CuO NPs (∼5 nm) effectively inhibited *Aspergillus flavus*, reducing aflatoxin levels, enhancing seed germination, and mitigating risks to storage and food safety	Food preservation	Ngwenya et al. (2025)
	Wet-chemical synthesis: ZnO2-Cu@RB NPs	ZnO2-Cu@RB NPs eliminated *Streptococcus* mutans (>8.0 log_10_ CFU mL^-1^), attenuate acid production, and inhibit EPS synthesis	Dental plaque treatment	Zhang et al. (2022a)
	Green synthesis:CuO NPs produced using Haloxylon salicornicum aqueous extract	CuO NPs (∼70 nm; spherical and hexagonal) enhanced tobacco growth and reduced AMV viral load by 97% upon foliar application, highlighting their potential against plant viral infections	Agriculture: antiviral agents	Aseel et al. (2024)
	Green synthesis:Cu NPs produced using Clitoria ternatea leaf extract	Spherical Cu NPs (∼60 nm) exhibited potent inhibitory activity against *B. subtilis*, *E. coli*, *Aspergillus niger*, and Sesbania mosaic virus (SeMV)	Agriculture: antimicrobial agents	Shaik et al. (2025)
	Wet-chemical synthesis: Cu NPs	Cu NPs (∼90 nm) exhibited potent inhibitory activity against *Xanthomonas*, *Bacillus*, *Pseudomonas*, and *Clostridium spp*.	Agriculture: antimicrobial preservation	Arya et al. (2024)
	Green synthesis:CuO@Fe2O3 NPs produced using Portulaca oleracealeaves extract	Photocatalytic CuO@Fe2O3 NPs (∼50 nm) exhibited antiviral activity against SARS-CoV-2 and enable light-driven antibiotic degradation	Agriculture: antiviral agents and environmental remediation	Legmairi et al. (2023)
	Deep eutectic solvent (DES) technology: Cu NPs	Cu NPs (∼100 nm) generated ROS, inducing cell death and exhibiting >93% antimicrobial efficacy against *S. aureus*, *E. coli*, *C. albicans*, H1N1, HCoV-OC43, and VSV.	Disinfectants	Długosz et al. (2025)
	Seed-mediated selective deposition:Au-CuS NPSs	The Au-CuS NPSs (∼5 nm) effectively inactivated NV at a minimal concentration of 0.083 μM	Disinfectants	Broglie et al. (2015)
	Electrodeposition technique: CuO + Cu_2_O nano-coating	The nanostructured coating (∼560 nm) exhibited pronounced inhibitory activity against *S. aureus* and *E. coli*	Antimicrobial surfaces	Kusior et al. (2024)
	Hydrothermal synthesis: Cu and Cu–Zn nanowires inks	The fabricated coating (∼60 nm) exhibited superior inhibitory activity against SARS-CoV-2 compared to pure copper surfaces	Antimicrobial surfaces	Pan et al. (2022)
	Wet-chemical synthesis: GO–CuI NPs	The spherical GO–CuI NPs (∼30 nm) exhibited potent bactericidal activity against *E. coli* and *S. aureus*	Antimicrobial surfaces	Hemmat et al. (2023)
	Protein-based nanocoating + metallization: copper–rhodopsin biomimetic cicada wing nanostructure	The biomimetic nanocoating exhibited superior antimicrobial and antiviral activity with reduced cytotoxicity compared to pure metal coatings	Antimicrobial surfaces	Kryuchkov et al. (2022)
	Spray-coating: Cu nanoparticles–polyurethane hybrid solution fabrication of antimicrobial surfaces	The spray-coating inactivated 99% of SARS-CoV-2 within 30 min	Antimicrobial surfaces	Foffa et al. (2022)
	Spin-coating: CuO-based self-disinfecting passivation coatings	CuO NPs (∼20 nm) exhibited potent inhibitory activity against *S. aureus* and HSV-2	Antimicrobial surfaces	Mannai et al. (2025)
	Sol–gel synthesis: photocatalytic Cu-doped TiO_2_ NPs	Under light activation, the Cu-doped TiO_2_ NPs inactivated MRSA, *K. pneumoniae*, feline calicivirus, and HCoV-229E	Antimicrobial surfaces	Campbell et al. (2024)
	Microwave-assisted hydrothermal synthesis + thermal treatment: Cu_3_Mo_2_O_9_ semiconductor nanoparticles (CMO)	The calcium alginate films loaded with CMO released Cu^2+^ and Mo^6+^ ions and generates ROS even in darkness, exhibiting potent activity against *P. aeruginosa*, *Mycobacterium*, phage phi 6, and MS2	Antimicrobial surfaces	Assis et al. (2024)
	Wet chemical synthesis: Ag–Gr, Cu–Gr, and Ag–Cu–Gr NPs	Cu–Gr NPs (150–250 nm thick) exhibit best anti-H1N1 activity	Antimicrobial surfaces	Das Jana et al. (2020)
	Protein self-assembly:A novel copper-binding peptide coating	The 6 nm transparent nanocoating releases Cu^+^ ions and H_2_O_2,_ conferring strong antimicrobial and antiviral efficacy	Antimicrobial surfaces	Boas and Reches (2021)
	Wet-chemical synthesis: Cu_2_O–zeolite NPs	Nanoclusters (50–100 nm) exhibited potent inhibitory activity against *E. coli*, *S. aureus*, PEDV, and SARS-CoV-2, and can be applied as transparent antimicrobial coatings on PET or PVC films	Antimicrobial surfaces	Jampa et al. (2022)
	Wet-chemical synthesis: CuM coated nanofilm based on Cu_2_O and MIL-53 (Al) composite	CuM exhibited potent activity against *E. coli*, *S. aureus*, HCoV-229E, influenza A virus, and EV71, and can be fabricated into safe, low-cost antimicrobial transparent films	Antimicrobial surfaces	Gamonchuang et al. (2024)
	Biotemplating approach: Cu nano-fibres	Copper nanofibres with ∼4 nm nanoflower morphology achieved >99.9% antiviral efficacy against HCoV-229E, SARS-CoV-2, and HRV-14, retaining activity after repeated high-temperature laundering and over 12 months of storage	Textile	Losada-Garcia et al. (2023)
	Green synthesis: Cu_2_O NPs produced using camu-camu extract	Cu_2_O NPs (∼10 nm, quasi-spherical) reduced SARS-CoV-2 viral load by 99.79%	Textile	Asmat-Campos et al. (2023)
	Green synthesis:Cu NPs produced using the Aqueous Extract of *Lonicera japonica* Thunb	Spherical Cu NPs (∼5 nm) exhibited antimicrobial activity against *Aspergillus niger*, *S. aureus*, *E. coli*, and *C. albicans*	Textile	Asmat-Campos et al. (2023)
	Wet-chemical synthesis: CuI NPs	Spherical CuI NPs (18–20 nm) exhibited potent antibacterial and antiviral activities, along with effective biofilm inhibition	Textile and self-sanitizing fabrics and wound dressings	Avatefi et al. (2024)
	Electrospray technology: Copper nanoparticles coated polyurethane membrane fibers	The copper fibers (∼650 nm) exhibited potent inhibitory activity against *E. coli* and SARS-CoV-2	Textile industry and medical protective equipment	Al Kayal et al. (2023)
	High-pressure jet milling: micron-sized CuI particles reduced to the nanoscale	CuI NPs (∼160 nm) exhibit potent anti-H1N1 activity at a minimal effective concentration of 17 μg/mL	Textile industry and medical protective equipment	Fujimori et al. (2012)
	Wet-chemical synthesis (CuNPs)+UV/O_3_ surface modification:Cu NPs-based melt-blown coatings	Spherical CuNPs (90–200 nm) exhibited potent inhibitory activity against SARS-CoV-2, *S. aureus*, and *E. coli*	Textile industry and medical protective equipment	SadrHaghighi et al. (2023)
	*In situ* synthesis: Cu_2_O NPs coated cotton fibers	The Cu_2_O NPs coated cotton fibers released only 19% of their copper content after 50 wash cycles and exhibited potent antimicrobial and antiviral activity against *K. pneumoniae*, *E. coli*, *S. aureus*, *C. albicans*, and HCoV-229E	Textile industry and medical protective equipment	Hillyer et al. (2022)
	*In situ* synthesis: CuO NPs coated fibers	The CuO NPs coated fibers exhibited strong biocidal activity against both SARS-CoV-2 and *E. coli*	Textile industry and medical protective equipment	Román et al. (2022)
	Electrospinning: Cu-loaded PCL/PVP nanofibers	The Cu-loaded fibers achieved 99.99% inactivation of coronaviruses	Textile industry and medical protective equipment	de Moraes Segundo et al. (2024)
	3D printing: Copper-loaded nanofibers PLA/Cu NPs	Spherical Cu NPs (80–100 nm) exhibited potent virucidal activity against MHV-3 and HAdV-2	Textile industry and medical protective equipment	Silva Dias et al. (2025)
	Wet-chemical impregnation: nano CuO-coated cotton fibers	CuO NPs (∼20 nm) with hydrophobic ellipsoidal morphology exhibited potent inhibitory activity against *E. coli* and HAdV-B	Textile industry and medical protective equipment	Hussain et al. (2023)
	Dip-coating + spray deposition:Hybrid alginate–copper sulfate textile coating	The coated fabric achieved 99.99% inactivation of MHV-3 coronavirus	Medical protective equipment: face masks	Bataglioli et al. (2022)
	Vacuum deposition: Cu_2_O-coated polypropylene filter	A 20 nm Cu_2_O nanofilm uniformly coated on KF94 mask fibers reduced SARS-CoV-2 viral load by 75%	Medical protective equipment: face masks	Jung et al. (2021)
	Electrospinning: CuO/PAN composite nanofibers	CuO/PAN (∼40 nm) exhibited potent inhibitory activity against *E. coli* and *S. aureus*	Medical protective equipment: face masks	Hashmi et al. (2019)
	Phase inversion: Cu nanoparticles featuring a mixed-valence surface composition of Cu, Cu_2_O, and CuO	Spherical Cu NPs (5–8 nm) exhibited potent inhibitory activity against *S*. *aureus*, *E*. *coli*, *P*. *aeruginosa*, and enveloped (phi6) and non-enveloped RNA (MS2) and DNA (T4) viruses	Medical and public health: paints and coatings	Nag et al. (2024)
	Wet-chemical synthesis: Cu_2_O NPs	Cu_2_O NPs (∼250 nm) achieved >97.8% inhibition against SARS-CoV-2	Medical and public health: paints and coatings	Purniawan et al. (2022)
	Sonochemical deposition: CuO coated filter	The 150 nm leaf-like CuO coating exhibited potent inhibitory activity against *E. coli*, *S. aureus*, H1N1 influenza, and SARS-CoV-2 variants	Air filtration	Perelshtein et al. (2022)
	Melt-blown technique: CuO nanoparticles coated polypropylene fibers	The CuO-based nanofiber air filter exhibited potent virucidal and bactericidal activity against SARS-CoV-2, *P. aeruginosa*, *A. baumannii*, *Salmonella* spp., and *E. coli*	Air filtration	Mekapothula et al. (2024)
	Flame aerosol deposition: CuO NPs	CuO NPs (∼10 nm) exhibited potent anti-SARS-CoV-2 activity	Air and water filtration	Merkl et al. (2021)

### 5.1 Healthcare applications

Hospitals, as dense reservoirs of pathogenic microorganisms, are especially vulnerable to nosocomial infections driven by MDR bacteria and epidemic-prone viruses. Copper-containing materials—particularly copper nanoparticle coatings—have been widely incorporated into clinical environments, including medical implants, wound dressings, dental coatings, and personal protective equipment (PPE) (Woźniak-Budych et al., 2023). Self-sanitizing surfaces coated with copper, such as hospital beds, door handles, and elevator buttons, significantly reduce pathogen survival and limit cross-contamination (Jabłońska-Trypuć et al., 2022). Copper–titanium alloys helped prevent postoperative infections (Liu et al., 2014; Zhuang et al., 2021), and copper-embedded dressings inhibited biofilm formation and combat MDR pathogens (Avatefi et al., 2024; Al-Habeeb and Al-Bishri, 2024). Copper-containing hydrogels are being explored for chronic wound healing, including diabetic ulcers (Astaneh and Fereydouni, 2024). Additionally, copper nanoparticles inhibit the proliferation of *Streptococcus* mutans, thereby reducing dental plaque formation (Zhang Y. et al., 2022). Copper-based filtration fibers have also proven effective in the sterilization of air and water in clinical settings (Vincent et al., 2016; Jung et al., 2022; Dixit et al., 2025). Conventional PPE offers only passive protection and poses a risk of secondary transmission during disposal. In contrast, copper-coated masks and garments actively reduce microbial burden, lowering the risk of transmission (Cortes and Zuñiga, 2020; Zhang S. et al., 2022). Notably, copper modulates immune cell functions—including helper T cells, B cells, neutrophils, natural killer (NK) cells, and macrophages—thereby potentially enhancing host antiviral defenses. This immunomodulatory effect addressed a role for copper-based nanotherapeutics in infectious disease treatment (Li X. et al., 2023). Moreover, copper ions and complexes demonstrate adjuvant-like activity, positioning them as promising candidates for vaccine formulation (Abate et al., 2022). As antibiotic resistance escalates, copper nanomaterials also emerge as viable antibiotic alternatives (Crisan et al., 2021; Parvin et al., 2025).

### 5.2 Public and environmental applications

The COVID-19 pandemic has catalyzed demand for antimicrobial surfaces in high-traffic public areas such as hospitals, airports, transportation hubs and office buildings. Copper coatings drastically reduce the viability of viral and bacterial pathogens on surfaces, offering a practical solution for infection control (Hutasoit et al., 2020). These coatings can be applied as sprays or adhesive films, enabling long-lasting antimicrobial protection. High-power magnetron-sputtered Ta-Cu coatings on titanium alloys demonstrated tunable antibacterial activity—most notably in the TaCu-2 sample annealed at 600°C—by optimizing copper content and thermal treatment to effectively combat implant-associated pathogens such as *E. coli* and *P. aeruginosa* (Azamatov et al., 2025). Gas dynamic spray deposition of copper onto ABS plastic yields a durable antimicrobial coating for high-touch surfaces such as switch buttons, reducing microbial contamination by 2.7-fold over 22 weeks (Emelyanenko et al., 2024). Sprayable antimicrobial coatings comprising silver-loaded thiol-functionalized mesoporous silica nanoparticles (MSN-SH) immobilized on stainless steel via polyelectrolyte primers exhibit potent, broad-spectrum efficacy against bacteria and fungi under dry conditions (Bernardino et al., 2025). A pH-responsive polycaprolactone–copper peroxide (PCL-CuO_2_) composite coating, fabricated via suspension flame spraying, enables controlled Cu^2+^ and H_2_O_2_ release under acidic conditions, achieving >99.99% antibacterial efficacy against *E. coli* and *S. aureus, highlighting its potential for biomedical antimicrobial surfaces*. (Cui et al., 2024). Innovations in superhydrophobic nanocoatings further inhibit microbial adherence, improving surface cleanliness. Electrodeposited copper surfaces coated with Teflon exhibit robust superhydrophobicity and enhanced condensation heat transfer—improving efficiency by approximately 78% and maintaining performance under mechanical stress far better than nanoneedle-structured CuO (Park et al., 2022). Addressing concerns around single-use PPE, reusable copper-infused masks have been developed that maintain breathability while actively neutralizing pathogens, thereby reducing environmental burden and operational costs (Hadinejad et al., 2023; Zhou J. et al., 2020). Zinn et al. developed a self-sterilizing copper material that rapidly inactivates a broad spectrum of pathogens within 30–60 s, offering residue-free, long-lasting antimicrobial protection ideal for integration into PPE and high-touch surfaces (Zinn et al., 2021). Jung et al. engineered a 20 nm copper film on polypropylene filters via vacuum deposition and oxygen ion pretreatment, enabling KF94 masks to inactivate over 75% of SARS-CoV-2 while retaining high filtration performance, advancing next-generation protective materials (Jung et al., 2021). Moreover, antimicrobial copper-based paints and coatings for walls and glass surfaces are under active development, offering the potential to reduce disinfection frequency and significantly lower labor costs in facility maintenance. Early dark-toned formulations have evolved into light-colored or transparent variants, such as the diatomite/Cu_2_O/CPT composite by Zou et al. (2024), and the transparent glass-ceramic copper coatings developed by Culpepper et al. (2025), both exhibiting strong and broad-spectrum antiviral activity with commercial promise. Golovchak et al. developed a durable, low-cost Cu–Sr phosphate glass that eradicates *S. aureus* within 24 h at low concentrations while remaining biocompatible, offering broad potential for antimicrobial medical and public-use coatings (Golovchak et al., 2025).

### 5.3 Textile applications

Textile-based vectors of disease transmission are also a concern. Traditional antimicrobial textiles suffer from rapid functional degradation caused by repeated washing and perspiration exposure, and may also pose toxicity risks (Broadhead et al., 2021). Nanotechnology now allows for the durable integration of copper nanoparticles into fabric fibers, preserving antimicrobial efficacy after more than 20 washing cycles (Mohamed et al., 2021; Hillyer et al., 2022). This advancement has enabled the development of medical textiles, including antimicrobial gauze, bandages, surgical gowns, and wipes. Cellulose-based fabrics, inherently susceptible to bacterial contamination, have been transformed into superhydrophobic, antibacterial textiles with enhanced resistance to pathogen adhesion through surface micro/nanostructuring and chemical modifications (Zhou H. et al., 2023; Alashkar et al., 2024). Priyanka et al. engineered hydrophobic nanocoated cotton fabrics by integrating mussel-inspired polydopamine, graphene oxide, and copper compounds, resulting in textiles that effectively repel fluids and inhibit bacterial growth (Prabhakar et al., 2022). Han et al. fabricated superhydrophobic copper nanoparticle-coated cotton fabrics via sonochemical deposition in alkaline media, achieving 145° water contact angles and effective antibacterial performance through Lotus-inspired micro/nano-scale surface structuring (Han and Min, 2020). Chen et al. fabricated superhydrophobic copper-coated cotton fabrics featuring micro/nano coral-like architectures via self-assembly and spray deposition, achieving a water contact angle of 161° alongside remarkable abrasion resistance, corrosion durability, and intrinsic conductivity (Chen et al., 2022). Investigating the influence of weave structure on inkjet printing quality, Sandu et al. demonstrated that electroless copper-plated textiles activated by inkjet-printed Cu/Ag catalysts along the weft exhibited durable antipathogenic efficacy—including virucidal activity against HCoV-OC43, HCoV-229E, influenza A (H1N1), and rotavirus A—while maintaining low cytotoxicity and year-long antibacterial stability (Sandu et al., 2025). Muhammad-Amir et al. further revealed that green-synthesized copper-treated cotton fabrics showed ∼60% higher tensile strength in the warp and ∼20% in the weft, with improved dye uptake and fastness, highlighting the key role of dyeing direction and fiber orientation in enhancing textile performance (Amir et al., 2023). To further enhance the safety and wearability of copper nanoparticles embedded in textile fibers, recent studies have explored strategies such as core–shell encapsulation (Komeily-Nia et al., 2019; Kuo et al., 2024), surface passivation with biocompatible polymers to reduce cytotoxicity (Matijaković Mlinarić et al., 2024; Calais et al., 2024), integration of metal–organic frameworks (MOFs) for multifunctional wearable systems (Eagleton et al., 2022; Eagleton et al., 2023; Xiao et al., 2024), precise nanoparticle immobilization through covalent bonding or *in situ* synthesis within fiber matrices, aimed at minimizing environmental leaching and dermal exposure (Xiao et al., 2024; Jalali et al., 2024; Huang et al., 2022; Tan X. et al., 2024; Bayisa et al., 2024; Zhao Z. et al., 2024), as well as green nanoengineered fabrics for improved biocompatibility (Meganathan and Ramalingam, 2024; Yu et al., 2025; Asmat-Campos et al., 2023). Additionally, time-dependent release kinetics (Ferrer-Vilanova et al., 2025), cytocompatibility assays on human skin cell lines (Świerczyńska et al., 2024), and long-term stability (more than 50 washing cycles) under washing and wear conditions have become standard evaluation metrics (Wen et al., 2024), ensuring both efficacy and biosafety for clinical and consumer applications.

### 5.4 Food packaging applications

Antimicrobial packaging plays a critical role in ensuring food safety by preventing microbial contamination and extending shelf life. Saravanakumar et al. developed a cellulose nanowhisker–sodium alginate (CNW–SA) composite film loaded with CuO nanoparticles (NPs), which exhibited potent antibacterial activity against *S*. *aureus*, *E*. *coli*, *Salmonella* spp., *Candida albicans*, and *Trichoderma* spp. (Saravanakumar et al., 2020). Zhao et al. developed polylactic acid (PLA)/halloysite–Cu^2+^ composite nanofiber membranes exhibiting superior biocompatibility, mechanical robustness, thermal stability, hydrophobicity and antibacterial efficacy, markedly enhancing strawberry preservation (Zhao X. et al., 2024). Shi et al. demonstrated that a nanocopper/polypropylene composite conferred enhanced antioxidant and antimicrobial properties, significantly extending the freshness and shelf life of packaged foods (Shi et al., 2021). The green-synthesized copper nanoparticles offer an economically feasible and non-toxic approach in food packaging. Kumari et al. demonstrated that Argemone mexicana–mediated green synthesis of Cu NPs within κ-carrageenan films produces biodegradable packaging with enhanced thermal stability, mechanical robustness, water-vapour and UV-barrier performance, and potent antibacterial activity against *S. aureus* and *E. coli*, extending grape and cottage cheese shelf life to 12 and 7 days, respectively (Kumari et al., 2024). Moldovan et al. engineered PLA/Proviplast composites incorporating 0.5–1.5 wt% grape pomace or 2–8 wt% PEG600-stabilized Cu particles, which function as bioactive plasticizers—reducing Tg, Tcc and Tm and modestly lowering thermal stability—while significantly boosting elongation at break and modulus, thereby creating flexible, eco-functional materials that valorize agricultural waste for sustainable active food packaging (Moldovan et al., 2024). Che et al. developed quercetin–copper nanoparticles with strong antioxidant and antibacterial activity, which effectively reduced weight loss and extended the shelf life of Shine Muscat grapes (Che et al., 2025). Although copper nanoparticles confer functional benefits to food packaging, their potential migration into food poses safety risks, warranting comprehensive future safety evaluations before commercial adoption. Copperprotek USA’s FDA/FSIS GRAS-approved 100% copper microparticles—the first copper salt-based additive cleared for food-packaging applications—are entering industrial-scale testing with a major U.S. packaging firm, enabling integration into animal-based products from 2025 and marking a milestone in antimicrobial, shelf life–extending packaging technology (Copperprotek, 2025). Moreover, in food processing environments, non–food-contact surfaces—such as drains, transport carts and equipment casings—harbour aerosol-transmissible pathogens like norovirus and hepatitis a virus, which copper-based coatings can efficiently inactivate to prevent indirect contamination (Camacho et al., 2023).

### 5.5 Agriculture and aquaculture applications

Beyond healthcare and food safety, copper-based systems are increasingly applied across agriculture, aquaculture, marine environments, and electronic industries. In animal husbandry, copper salts and nanoparticle formulations are used as bactericides, algicides, insect repellents, and preservatives (Kanhed et al., 2014). Möhrke et al. showed that twin-wire arc-sprayed copper coatings—using compressed air or nitrogen—achieved a 99% reduction in pathogenic bacteria common in broiler farming (*E. coli*, *S. aureus*, *Escherichia cecorum*) compared to uncoated steel, with post-treatments such as cold plasma and TIG arc further enhancing antibacterial efficacy and durability (Möhrke et al., 2024). Cu NPs—serving as growth promoters, antioxidants, and antibiotic alternatives—hold promising potential for broad biotechnological applications (Sharif et al., 2021; Qadeer et al., 2024; Nechitailo et al., 2025). However, the reported toxicity of Cu NPs necessitates further studies to elucidate their mechanistic effects and safety in animal husbandry (Sabry et al., 2021).

In aquaculture, copper-coated or alloyed equipment—tanks, pipes, filters, enclosures—minimizes biofouling and cross-species transmission of pathogens, aiding in the containment of AMR. Liu et al. reported that *in situ* growth of Cu-MOF films on alkali-heat-treated Ti-6Al-4V produced bioactive coatings with strong antibacterial and algicidal activity (Liu and Gao, 2024). Ponurko et al. reported that copper-containing glassy phosphate compositions (CGPCs) form continuous phosphate films and release Cu^2+^ ions in aqueous environments, synergistically inhibiting microbial growth by blocking oxygen access and disrupting biological activity, highlighting their potential for broad-spectrum water treatment applications (Ponurko et al., 2023). Ilkhas et al. developed a Cu-doped ZnO/reduced graphene oxide nanocomposite synthesized in one step that efficiently degrades antibiotics and inactivates resistant bacteria in shrimp aquaculture water (Ilkhas et al., 2024).

In crop science, copper-based nanomaterials serve as both essential micronutrients and antimicrobial agents. These nanomaterials represent a promising avenue for improving crop yield and managing plant diseases, functioning as nanofertilizers, nanoregulators, nanostimulants, and nanopesticides to enhance plant growth, stress resistance, and seed germination. In particular, the green synthesis of copper-based nanoparticles enables environmentally sustainable agricultural strategies (Amin and Aziz, 2025). Martins et al. demonstrated that controlling Cu^2+^ ion release from CuO-based nanofertilizers using plant growth regulator–derived ionic liquids significantly enhanced photosynthetic efficiency, biomass accumulation, and CO_2_ capture in *Nicotiana tabacum*, highlighting the pivotal role of ion dissolution kinetics in the rational design of sustainable nanofertilizers (Martins et al., 2024). Notably, copper nanoparticles biosynthesized using endophytic fungi have been shown to possess strong biocidal activity and to stimulate plant innate immune responses, offering new biocompatible tools for advancing sustainable crop production (Selim et al., 2025).

### 5.6 Marine applications

In marine engineering, HEAs and copper-based coatings are widely utilized on ship hulls to mitigate biofouling, reduce hydrodynamic drag, and prevent corrosion (Yu et al., 2021; Kuptsov et al., 2023). Zhou et al. engineered Cu–Ag HEAs exhibiting enhanced Cu^+^/Cu^2+^ ion release, alongside superior mechanical strength, corrosion resistance, and broad-spectrum antimicrobial activity—achieving 99.9% bacterial inhibition and approximately 99% deactivation of SARS-CoV-2 (Zhou et al., 2024). Ding et al. reported that Cu_2_O-containing marine coatings based on poly (lauryl methacrylate)-*b*-poly (2-(N,N-dimethylamino)ethyl methacrylate) copolymers enabled controlled copper ion release, significantly improving both bactericidal and antifouling efficacy for sustainable marine applications (Wang et al., 2025). Li et al. functionalized three-dimensional porous Cu_2_O nanoparticles (3DNP-Cu_2_O/rGOx@R-Gel) to promote sustained Cu^+^ ion release, achieving potent antibacterial and antifouling performance while minimizing overall copper ion leaching. The incorporation of reduced graphene oxide (rGO) and R-Gel facilitated the *in situ* reduction of Cu^2+^ to Cu^+^ and enhanced system stability under marine conditions (Li H. et al., 2023). Furthermore, biomimetic copper nanostructures, mimicking naturally antimicrobial surface morphologies, have been developed to enhance antifouling efficacy and material durability, with potential applications extending across marine and biomedical domains (Ruggeri et al., 2024; Li et al., 2024; Chen et al., 2021). A notable example is a bioinspired shark-skin-like antimicrobial surface fabricated on titanium alloy via a single-step wire electrical discharge machining (WEDM) process, which achieved 93% bacterial inhibition, further enhanced to 98.4% after acid etching, along with excellent bioactivity—underscoring its applicability in marine environments (Zhang et al., 2023). Liu et al. fabricated thermally stable, wood-inspired copper surfaces using metallic glass templating techniques, achieving robust hydrophobicity and anti-icing performance under extreme environmental conditions. These surfaces highlight the potential of structurally engineered copper materials for long-term use in harsh marine and shipbuilding scenarios (Liu et al., 2021).

### 5.7 Electronic applications

Furthermore, copper-based coatings are increasingly integrated into antimicrobial glass and plastic surfaces of high-touch electronics—such as smartphones, laptops, and tablets—to mitigate microbial adhesion and reduce the risk of fomite-mediated infections (Boas and Reches, 2021; Gamonchuang et al., 2024). Tian et al. developed amine–carboxyl (AC) co-modified Cu-AC nanoparticles with high monodispersity and antioxidant capacity, which synergistically enhance the antibacterial, thermal, and mechanical properties of polypropylene composites, achieving up to 99% antimicrobial efficacy and offering broad potential in thermoplastic applications for frequently handled surfaces (Tian et al., 2024). Golovchak et al. reported a cost-effective and durable Cu-containing strontium-modified phosphate glass with potent antibacterial activity against *S. aureus*, highlighting its potential in antimicrobial glass technologies (Golovchak et al., 2025). Jiang et al. demonstrated that Cu^+^ -doped ion-exchanged glass exhibited an enhanced mechanical strength, and sustained antimicrobial activity via controlled surface incorporation of copper (Jiang et al., 2024). In addition to their antimicrobial utility, copper-based materials exhibit excellent catalytic activity, making them attractive candidates for environmental remediation (Bonthula et al., 2023). For instance, Vinothkanna et al. demonstrated that biogenically synthesized copper oxide nanoparticles derived from *Rubia cordifolia* bark extract possess potent antibacterial, antioxidant, larvicidal, and photocatalytic properties (Vinothkanna et al., 2023). Similarly, Kumar et al. developed Co/Cu-doped hematite nanoparticles using *Azadirachta indica* leaf extract, achieving tunable crystalline and magnetic properties alongside robust photocatalytic and antioxidant activity—emphasizing their promise as eco-friendly agents in environmental clean-up applications (Kumar et al., 2024).

### 5.8 Clinical and field trials

Several ongoing clinical and field trials are currently investigating the real-world efficacy of copper-based materials. These include studies on copper’s impact on antimicrobial resistance in ICUs (CUPRIC, NCT04873557), its role in wound healing (NCT01565798, NCT02351895, NCT03284749, NCT05215730), and its effectiveness in reducing healthcare-acquired infections in pediatric ICUs (NCT01678612). Additionally, copper’s use in agriculture is being tested in field trials targeting *Pseudomonas syringae* in Nicotiana tabacum production (Webb and Bailey, 2024). These trials provide valuable data supporting copper’s antimicrobial applications in clinical and public settings, as well as in agriculture.

## 6 Copper-based materials at the crossroads: challenges and future perspectives

Despite the well-established antimicrobial potency of copper and its derivatives, translating these capabilities into sustainable, safe, and effective real-world applications remains fraught with complexity (Kadiyala et al., 2018). At the core of this challenge lies the delicate balance between antimicrobial efficacy and biological safety—largely dictated by the release kinetics, oxidation states, and environmental stability of copper ions (Park et al., 2024; Martins et al., 2024; Shigetoh et al., 2023). While rapid ion liberation can enhance antimicrobial activity, it also accelerates corrosion, increases cytotoxicity, and undermines the structural integrity and longevity of the material (Luo et al., 2019; Cao et al., 2012). Accordingly, engineering copper-based systems with spatiotemporal control over ion release has emerged as a critical frontier. Innovative strategies—including high-entropy copper alloys, nanostructured microporous matrices, and surface-confined copper-based platforms—have achieved partial success in modulating ion flux. However, none have fully reconciled the efficacy–biosafety trade-off, particularly under physiologically relevant conditions (Zhou et al., 2024; Selvamani et al., 2020; Mitra et al., 2019).

From a materials engineering perspective, several factors dictate copper ion release rates in physiological and environmental conditions. Material composition, such as copper content in alloys or oxidation state, directly influences release kinetics, with Cu_2_O releasing Cu^+^ ions faster due to higher redox activity (Birkett et al., 2022; Park et al., 2024). Surface morphology, including high surface-area-to-volume ratios in nanoparticles or increased roughness via cold spraying, enhances ion diffusion by providing more active sites (Sousa et al., 2021; Razavipour et al., 2022; Ghezzi et al., 2022). Defect-rich CuZn nanoparticles, as shown by Lasemi et al., further accelerate release through increased surface reactivity (Lasemi et al., 2024). Environmental factors like acidic pH (e.g., in infected tissues) promote copper oxide dissolution, boosting ion release and antibacterial efficacy (Cui et al., 2024). However, organic matter, such as proteins or humic acids, can chelate ions, reducing bioavailability (Saravanakumar et al., 2020). Matrix design, including polymeric encapsulation or surface functionalization with glutamic acid, controls release rates and improves biocompatibility (Hall et al., 2024; Saravanakumar et al., 2020). Fabrication techniques like laser ablation or electrochemical deposition allow precise control over grain size and porosity, tailoring ion release for specific applications (Robinson et al., 2021; Patlejchová et al., 2023). These considerations enable optimized material design for sustained antimicrobial performance.

Environmental factors significantly influence copper ion leaching and antimicrobial efficacy. Acidic pH (e.g., in infected tissues) accelerates copper oxide dissolution, increasing Cu^2+^/Cu^+^ onions release and enhancing ROS production for >99.99% bacterial killing (Cui et al., 2024). Organic matter chelates ions, reducing bioavailability and efficacy, though nanotextured surfaces mitigate this via contact killing (Kuyukina et al., 2025). Higher temperatures increase ion release by enhancing oxidation kinetics, but excessive heat may alter oxide composition, reducing efficacy (Park et al., 2024). Context-aware applications, such as pH-responsive PCL-CuO_2_ coatings, release copper ions selectively in acidic microbial environments (e.g., wounds, biofilms), achieving high efficacy while sparing host tissues (Cui et al., 2024). Antifouling zwitterionic coatings reduce organic matter adsorption, maintaining ion release and contact-killing efficacy in physiological or environmental settings (Li H. et al., 2023).

Electrochemical deposition methods, long employed to fabricate copper coatings, underscore how subtle changes in electrolyte composition can profoundly influence surface topography, redox behavior, and antimicrobial performance (de Lima et al., 2024). Templated electrodeposition, in particular, enables the formation of nanodendritic architectures that exhibit enhanced contact-killing efficacy, attributed to increased surface roughness and the creation of localized ion-rich microenvironments. Although distinct in mechanism and outcome, alternative techniques such as thermal spraying, chemical vapor deposition (CVD), physical vapor deposition (PVD), and sputtering continue to face challenges related to interfacial instability—especially on polymeric and textile substrates—which limits their scalability for high-touch applications (Bharadishettar et al., 2021). Nanotextured surface contact killing contributes significantly to efficacy, independent of ion release. High-aspect-ratio structures mechanically rupture microbial membranes, causing cytoplasmic leakage (Bhatti and DeLong, 2023; Acharya et al., 2021). In physiological media, contact killing accounts for 20%–50% of efficacy, though organic matter adsorption may reduce effectiveness; high surface energy minimizes fouling (Ghezzi et al., 2022). In environmental media, laser-ablated CuO/Cu_2_O NPs achieve rapid pathogen inactivation (5 h) via contact killing (Hesabizadeh et al., 2023). In air interfaces, copper-coated surfaces achieve >90% bacterial inactivation through direct contact (Mekapothula et al., 2024). Selvamani et al. highlighted that hierarchical copper structures enhance contact killing against *E. coli* (Cao et al., 2012). Fabrication techniques like laser ablation and electrochemical deposition create high-aspect-ratio structures, optimizing antimicrobial performance (Hesabizadeh et al., 2023; Selvamani et al., 2020). These designs are particularly effective in dry environments where ion diffusion is limited, ensuring broad applicability across media (Perelshtein et al., 2022).

Copper-based nanoparticles, characterized by their high surface-to-volume ratios and potent bioactivity, were increasingly investigated as potential next-generation antimicrobial candidates (Mitra et al., 2019). However, their physicochemical instability, aggregation propensity, and uncontrolled ion release pose significant challenges to safe and effective deployment. Environmental factors—such as pH, ionic strength, temperature, and the presence of biological macromolecules or biofilms—profoundly influence nanoparticle behavior, complicating dose-response relationships and undermining *in vivo* predictability (Orta-Rivera et al., 2023). Advances in stimuli-responsive copper-based nanomaterials—including pH-sensitive hydrogels, enzyme-cleavable micelles, and photoactivated nanocomposites—offer the potential for localized, on-demand antimicrobial activity (Wu et al., 2024; Vippala et al., 2024; Li Y. et al., 2018; Hou et al., 2021; Zhou Y. et al., 2023; Guo et al., 2025; Zu et al., 2022). Polymeric supports like PLA, PCL, and hydrogels maintain copper ion release efficiency through matrix design and functionalization. Hydrogels’ high water content and tunable porosity enable sustained Cu^2+^ release over weeks, as shown by Zu et al. with copper peroxide–hydrogels (Zu et al., 2022). Surface functionalization stabilizes nanoparticles, preventing aggregation and ensuring consistent release (Woźniak-Budych et al., 2024). PLA–Cu composites retain ∼80% antibacterial activity after 10 washing cycles due to controlled diffusion (Moldovan et al., 2024). Repeated exposure may deplete ions, while hydrolytic degradation of PCL/PLA accelerates release over time (Cui et al., 2024). Organic matter fouling can reduce efficiency, but antifouling coatings mitigate this (Li H. et al., 2023). Polymeric supports face challenges like ion depletion and polymer degradation after repeated use (Cui et al., 2024; Moldovan et al., 2024). Core–shell nanoparticles, MOF-based coatings, and antifouling zwitterionic surfaces enhance durability by protecting copper and reducing fouling (Komeily-Nia et al., 2019; Li H. et al., 2023). Non-degradable polymers or cross-linked hydrogels can extend ion release longevity for biomedical and environmental applications (Woźniak-Budych et al., 2024). Nevertheless, clinical translation remains hindered by the lack of standardized fabrication protocols, poor batch-to-batch reproducibility, and insufficient pharmacokinetic and toxicological profiling (Woźniak-Budych et al., 2023; Zhong et al., 2022).

Toxicologically, copper’s Janus-faced nature—being both essential and potentially deleterious—presents a persistent paradox. Excessive accumulation of Cu^+^ or Cu^2+^ has been linked to hepatotoxicity, nephrotoxicity, mitochondrial dysfunction, and neurodegeneration, primarily via Fenton-like ROS generation and disruption of key metabolic pathways (Ringu et al., 2024). Despite increasing *in vitro* evidence, long-term *in vivo* assessments remain scarce. Specifically, chronic toxicity, immunogenicity, and biodistribution in relevant animal models are poorly characterized, hindering clinical translation. Furthermore, the oxidation-state dynamics of copper-based nanoparticles within complex biological matrices are poorly characterized, yet likely dictate both their antimicrobial efficacy and adverse biological outcomes (Fernández-García et al., 2024). Achieving strong antimicrobial activity while ensuring host cell compatibility requires tailored material design. Polymeric encapsulation (e.g., PLA, PCL, hydrogels) controls copper ion release, reducing cytotoxicity while maintaining efficacy (Pourmadadi et al., 2024; Saravanakumar et al., 2020; Guo et al., 2025). Zu et al. developed copper peroxide–hydrogels that release Cu^2+^ onions in acidic wounds, achieving >99.99% antibacterial efficacy with minimal fibroblast toxicity (Zu et al., 2022). Core-shell structures and Cu-MOFs provide sustained release, passing cytocompatibility tests on skin cells (Komeily-Nia et al., 2019). Surface charge modulation, such as positive amine groups or zwitterionic sulfobetaine, enhances bacterial targeting while minimizing host cell damage (Woźniak-Budych et al., 2024; Li H. et al., 2023). pH-responsive coatings release ions selectively in microbial microenvironments, sparing neutral host tissues (Cui et al., 2024). Doping with Ag/Zn or green synthesis with plant extracts further reduces required copper doses, enhancing biocompatibility (Kasi et al., 2024; Vinothkanna et al., 2023). Biocompatible copper coatings, such as PCL–CuO_2_ for wound dressings and Cu-MOFs for implants, achieve high antibacterial efficacy (>99.99%) with low cytotoxicity due to controlled ion release and pH-responsive designs (Cui et al., 2024; Zu et al., 2022). Sulfobetaine-stabilized Cu_2_O nanoparticles selectively target bacteria, minimizing mammalian cell toxicity for medical applications (Woźniak-Budych et al., 2024).

To ensure translatability, preclinical studies should adopt standardized assays to balance efficacy and safety. Efficacy metrics include ROS quantification to assess oxidative stress (Kuyukina et al., 2025), ZOI for antibacterial potency (Pricop et al., 2025), MIC/MBC for dose optimization (Siddique et al., 2024), and biofilm disruption assays (Sedighi et al., 2024; Wang et al., 2017b; Wang et al., 2021). Safety metrics include cytocompatibility such as MTT assays on fibroblasts for Cu_2_O-modified cellulose, hemocompatibility through hemolysis assays, genotoxicity via comet assays, and environmental toxicity including Daphnia magna tests. Testing under physiological conditions including pH, ionic strength, and biofilm presence aligns with real-world applications, as per ISO 22196:2011 and ISO 7581:2023 (Bento de Carvalho et al., 2024; Maitz et al., 2024). Multi-species testing against MRSA, *E. coli*, and *C. albicans* ensures broad-spectrum efficacy (Saravanakumar et al., 2020), while long-term stability tests such as those conducted after repeated microbial challenges confirm durability (Mohamed et al., 2021). These protocols bridge *in vitro* and clinical outcomes.

Optimization of nanoparticle design—including precise control over morphology, particle size distribution, crystallinity, and surface functionalization—is critical to balancing antimicrobial potency with colloidal stability and biocompatibility. While smaller particles exhibit enhanced reactivity, they are also more prone to aggregation and rapid systemic clearance. In contrast, larger particles may sediment quickly or display diminished bioactivity. Strategies such as smart dispersants, surface PEGylation, and ligand-assisted self-assembly have shown promise in mitigating these limitations (Mondal et al., 2024; Bregnocchi et al., 2022). Green synthesis approaches—leveraging plant extracts, bacteria, fungi, or biopolymers—have emerged as eco-compatible alternatives to conventional chemical methods, but remain constrained by batch-to-batch variability, low yields, and limited control over particle morphology. Green synthesis using biological templates such as plant extracts and microbes faces reproducibility challenges due to variability in phytochemical or biomolecule content (Javid-Naderi et al., 2025). Standardized extraction protocols including solvent and pH control along with biomolecule characterization through HPLC and GC-MS improve consistency, as shown by Nkosi et al. with *P. mirabilis* bioflocculants (Nkosi et al., 2025). Kumari et al. achieved uniform Cu NPs (20–30 nm) using κ-carrageenan films (Kumari et al., 2024). Scalability is feasible with plant extracts such as Rubia cordifolia due to their abundance (Vinothkanna et al., 2023), while microbial synthesis using fungi benefits from bioreactor optimization (Chaerun et al., 2022). Alternative templates like Terminalia bellirica or Pantoea agglomerans offer comparable reproducibility with standardized protocols (Selim et al., 2025; Yugandhar et al., 2018; Rani et al., 2025; Viswadevarayalu et al., 2016). Advances in automated extraction and bioreactors enhance industrial scalability (Hosseingholian et al., 2023). Green synthesis reproducibility is limited by biological variability, requiring standardized protocols for consistent nanoparticle characteristics (Javid-Naderi et al., 2025).

Hybrid nanocomposites—particularly those incorporating copper with graphene derivatives, metal–organic frameworks (MOFs), or biodegradable polymers—have emerged as promising platforms for simultaneously mitigating toxicity and enhancing antimicrobial efficacy (Lv R. et al., 2022; Avatefi et al., 2024; Huang et al., 2022; Ilkhas et al., 2024; Guo et al., 2025; Elmehrath et al., 2024; Balcucho et al., 2020). For instance, superhydrophobic copper–graphene coatings not only resist microbial adhesion but also retard corrosion and minimize ion leaching, providing a dual advantage in safety and performance (Sulthana et al., 2024). Likewise, Cu–MOF architectures facilitate encapsulation-based ion buffering, enabling sustained and microbe-responsive ion release tailored to environmental microbial burden (Davoodian et al., 2025; Gwon et al., 2021). In the case of copper-graphene heterostructures, antimicrobial activity is enhanced through a multi-target approach. Graphene’s sharp edges mechanically rupture bacterial cell walls, while its high surface area prevents copper nanoparticle aggregation, improving ion release efficiency (Lv R. et al., 2022). Copper ions bind to membrane phospholipids, increasing permeability, and trigger ROS production via Fenton-like reactions, leading to lipid peroxidation and protein/DNA damage (Tan et al., 2017; Tsvetkov et al., 2022). Under near-infrared irradiation, copper–graphene composites generate localized heat, denaturing microbial proteins and enhancing contact-killing efficacy (Sulthana et al., 2024). On the other hand, Copper–zinc oxide heterostructures combine ZnO’s photocatalytic superoxide and hydroxyl radical production with copper’s ROS, amplifying oxidative stress (Guo et al., 2021; Ngwenya et al., 2025). ZnO’s positive surface charge enhances adhesion to bacterial membranes, facilitating copper ion penetration, which disrupts intracellular metabolic pathways like the TCA cycle (Tan et al., 2017; Ngwenya et al., 2025). Ngwenya et al. demonstrated superior antifungal activity of CuO-ZnO hybrids against Aflatoxin B1, attributed to synergistic ROS and metal ion effects (Ngwenya et al., 2025). These heterostructures outperform commercial copper nanoparticles by reducing aggregation and adding photothermal/photocatalytic mechanisms, minimizing resistance development (Lv R. et al., 2022; Guo et al., 2021).

Material design can also target cuproptosis-like pathways by addressing Gram-positive and Gram-negative differences. For Gram-positive bacteria, Cu_2_O nanoparticles maximize ROS production to overcome thick cell walls (Park et al., 2024). Doping with ZnO or Ag enhances ROS, as seen in CuO-ZnO hybrids (Ngwenya et al., 2025). For Gram-negative bacteria, efflux pump inhibitors disrupt resistance, enhancing cuproptosis (Glišić et al., 2016). Additionally, surface functionalization with positive charges such as glutamic acid improves adhesion to LPS, increasing copper delivery (Hall et al., 2024). Moreover, Hypoxia-modulating frameworks, like MnO_2_-loaded copper systems, shift biofilms to aerobic respiration, amplifying susceptibility (Luo et al., 2024). These strategies optimize copper-based materials for broad-spectrum efficacy.

Recent studies highlight several novel mechanisms underlying the antimicrobial efficacy of copper-based nanoparticles. These include redox cycling that enhances oxidative stress through Fenton-like reactions, leading to lipid peroxidation and nucleic acid damage (Warnes et al., 2012; Li W. et al., 2018; Ali et al., 2025). Shape-controlled CuNPs, such as nanocubes, improve biofilm penetration by disrupting QS and extracellular polymeric substances (EPS) in *P. aeruginosa* (Desai et al., 2021; Glišić et al., 2016; Liu et al., 2024). Additionally, CuNPs with photothermal and enzyme-mimetic properties amplify antimicrobial effects through localized heating and ROS production (Zhao et al., 2021; Ali et al., 2021; Ali et al., 2020). These advancements underscore the potential of optimizing nanocopper in combating AMR and viral pathogens, though further clinical validation is essential.

From a translational perspective, one of the most pressing bottlenecks lies in the integration of copper-based antimicrobials into existing public and clinical infrastructure. While solid copper and its alloys are effective, their high cost and susceptibility to oxidation limit feasibility for large-scale deployment. In contrast, copper-based antimicrobials offer a low-cost, scalable alternative, but often exhibit deep coloration, poor chemical stability, weak substrate adhesion, short service lifespans, and limited material compatibility (Zou et al., 2024; Graham et al., 2024). Advanced strategies such as surface-grafted antimicrobial moieties, plasma-assisted anchoring, and 3D-printed hierarchical architectures represent next-generation solutions to these challenges (Gautam et al., 2024; Silva Dias et al., 2025; Hirao et al., 2024; Benčina et al., 2021; Zhang et al., 2024). In textile applications, key obstacles include wash durability, mechanical abrasion resistance, dermal toxicity, breathability-waterproof balance, and personal thermoregulation. Innovations such as *in situ* synthesis during fiber spinning, covalent immobilization of copper-based nanoparticles, biomimetic superhydrophobic coatings, and integration with moisture-responsive polymers offer promising routes to enhance durability and skin compatibility (Chen et al., 2024; Román et al., 2022; Jian et al., 2025; Han et al., 2018; Yang et al., 2021; Li X. et al., 2021). In food packaging applications, the main concern of copper materials is safety. Copper-based nanomaterials in food packaging, such as Copperprotek’s GRAS-approved microparticles (GRN No. 1147), comply with FDA regulations (21 CFR 182) for safe use at a maximum level of 100 mg/m^2^ (Copperprotek, 2025; FDA. GRAS Notice No, 2024). The EU 2016/1416 had established upper limit of 5 mg/kg migration of copper from products into food simulants or food. Migration studies show nanocopper–polypropylene composites release <0.1 mg/kg copper in food simulants, below the EPA’s maximum contaminant level (1.3 mg/L) (Shi et al., 2021). κ-Carrageenan films release <0.05 mg/kg copper, meeting EFSA guidelines (Kumari et al., 2024). This benchmark serves as a safety reference; however, further toxicological studies are required to assess the risks associated with nanoparticle migration.

Beyond human health, the environmental footprint of copper-based nanoparticles demands urgent attention. Once released into natural ecosystems, these particles may accumulate in soil and aquatic matrices, exerting toxic effects on plants, invertebrates, and microbial biodiversity (Tortella et al., 2024; Wang and Liu, 2022). In agriculture, In agriculture, copper nanomaterials interact with soil organic matter and clay, which reduces the bioavailability of copper ions while enhancing their persistence in the environment. In aquatic sediments, CuO NPs persist at >50 mg/kg, reducing microbial diversity (Rajput et al., 2020). Bioaccumulation in invertebrates such as Daphnia magna and biomagnification in fish like zebrafish cause oxidative stress and reproductive toxicity at 0.1 mg/L (Wang and Liu, 2022). In soils, CuO NPs at 10–100 mg/kg alter microbial communities, reducing nitrogen-fixing bacteria (Tortella et al., 2024). Mitigation strategies include biomineralization such as Cu_2_S formation by Geobacter sulfurreducens and green synthesis using plant extracts like Rubia cordifolia to reduce toxicity (Kumari et al., 2024; Kimber et al., 2020). Long-term field studies are needed to assess ecological impacts.

Sublethal copper exposure can perturb nutrient cycles, disrupt microbial community composition, and drive the evolution of resistance. Mechanistically, copper exposure may enhance cell membrane permeability and promote the horizontal transfer of resistance genes among microbial populations (Kuyukina et al., 2025; Xu et al., 2023; Swiacka et al., 2023; Mesquita et al., 2023; Song et al., 2021; Liu et al., 2023). Microorganisms subjected to sublethal concentrations of copper-based nanoparticles may upregulate efflux pumps undergo genetic adaptations, or restructure biofilms to resist oxidative and ionic stress (Besaury et al., 2013; Virieux-Petit et al., 2022; Hajiagha and Kafil, 2023; El-Sherbiny et al., 2025). Specifically, low-dose copper exposure can induce AMR by upregulating specific gene expression pathways and efflux mechanisms. In bacteria like *E. coli* and *P. aeruginosa*, Copper homeostasis genes copA, cusA, and cueO are activated, with copA encoding a P-type ATPase for Cu^+^ export, cusA driving Cu^2+^ efflux via the CusCFBA system, and cueO oxidizing Cu^+^ to less toxic Cu^2+^ (Besaury et al., 2013; Virieux-Petit et al., 2022). Oxidative stress from copper-induced ROS triggers sodA superoxide dismutase and katG catalase expression to mitigate damage (Kuyukina et al., 2025). Biofilm formation genes pel and psl in *P. aeruginosa* are upregulated, increasing EPS production and copper sequestration (Kuyukina et al., 2025). Copper also promotes antibiotic resistance via conjugation, upregulating tra genes in plasmids like SXT/R391 (Zhang et al., 2019; Song et al., 2021). Efflux pumps, such as CusCFBA and MexAB-OprM, contribute to cross-resistance with antibiotics, complicating treatment (Besaury et al., 2013; Hajiagha and Kafil, 2023). Paradoxically, low-level copper exposure may stimulate biofilm formation via oxidative preconditioning in certain bacterial species, potentially exacerbating surface colonization and persistence (Kuyukina et al., 2025; Baker et al., 2010; Thakur et al., 2024). These findings underscore the need for a well-defined therapeutic window that maximizes antimicrobial efficacy while minimizing ecological disruption and resistance selection. Notably, copper-based nanoparticles engineered to interfere with QS pathways have exhibited potent anti-biofilm effects, offering a promising strategy to optimize function while mitigating resistance development (Wang et al., 2025; Lan et al., 2025). Monitoring resistance gene dissemination in environmental settings is critical to mitigate co-selection risks.

Finally, the absence of globally harmonized protocols and refined standards for evaluating the antimicrobial efficacy, toxicological risks, environmental persistence, and material durability of copper-based nanotechnologies has significantly impeded regulatory approval and commercial deployment (Hirao et al., 2024; Gupta et al., 2024; Kent and Vikesland, 2016). Current assays exhibit substantial variability in terms of test organisms, endpoints, and exposure conditions, complicating inter-study comparability. Establishing standardized metrics—including antimicrobial log-kill benchmarks, copper ion release rates, mechanical durability assessments, ecotoxicological profiles, biocompatibility assays, and biofilm inhibition performance—will be crucial for cross-sector adoption (Pourmadadi et al., 2024; Samarajeewa et al., 2021; Razavipour et al., 2022; Ren et al., 2021; Yao et al., 2018). While preliminary databases are available for bulk copper alloys, comprehensive repositories dedicated to copper-based nanomaterials remain conspicuously lacking (Gorsse et al., 2023; Ji et al., 2021), impeding systematic risk assessment and rational material design. Concurrently, the creation of open-access databases cataloguing structure–activity–toxicity relationships across diverse copper-based nanomaterials could substantially accelerate both risk governance and performance optimization.

In conclusion, while copper nanotechnology offers transformative potential for antimicrobial protection across healthcare, public infrastructure, and consumer applications, its trajectory depends critically on addressing fundamental scientific uncertainties, establishing standardized evaluation frameworks, and fostering interdisciplinary integration across materials science, microbiology, environmental science, and regulatory policy. Meeting these challenges with scientific rigor and long-term vision will be essential to determine whether copper-based materials can realistically contribute to the next-generation of antimicrobial strategies. Future research directions are increasingly focusing on enhancing long-term stability, safety, and scalability of copper-based systems. Promising avenues include bioinspired synthesis strategies, smart copper-responsive coatings, biodegradable copper–polymer nanocomposites, and synergistic formulations with plant-derived antimicrobials or probiotics.

## 7 Summary and concluding remarks

The COVID-19 pandemic has profoundly accelerated global interest in copper-based materials as frontline antimicrobial agents. Confronted with the urgent imperative to curb viral transmission—particularly that of SARS-CoV-2—researchers have rapidly expanded the design landscape of copper-containing nanostructures, generating a diverse array of antiviral coatings, surfaces, and nanocomposites. Beyond pandemic responsiveness, copper’s broad-spectrum efficacy, contact-based inactivation mechanism, and low propensity for resistance emergence underscore its value as a critical tool in the post-antibiotic era (Jung et al., 2021; Asmat-Campos et al., 2023; Purniawan et al., 2022; Perelshtein et al., 2022; Mekapothula et al., 2024).

**FIGURE 5:**
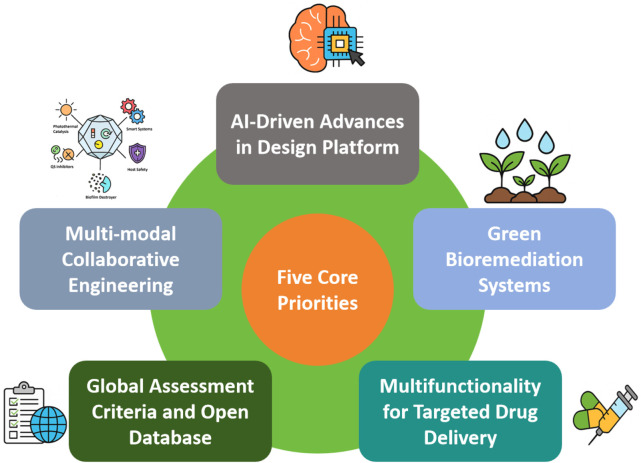
Anticipated research frontiers in copper-related antimicrobial materials.

Looking ahead, the next-generation of copper-centered research must tackle five interlocking priorities to fully realize its biomedical and environmental potential, as illustrated in Figure 5. First, the rational design of stable, intelligent, and economically scalable copper-based materials is imperative. This calls for atomic-level insights into structure–function relationships, particularly the role of grain boundary engineering, surface anisotropy, and nano–microstructural hierarchy in governing antimicrobial performance. Engineered surfaces—such as self-healing coatings, superhydrophobic films, redox-stable barriers, and recyclable nano-copper sprays—optimize the nanoparticle–cell interface and enable the controlled release of bioactive agents, providing a versatile platform for next-generation antimicrobial technologies. Artificial intelligence tools like MatterGen and M3GNet are accelerating the materials discovery pipeline by enabling predictive alloy formulation and coating optimization with significantly reduced experimental burden (Tan F. et al., 2024; Zeni et al., 2025; Merchant et al., 2023; Sharmila et al., 2024).

Second, the engineering of copper-based nanoparticles with tunable ion release kinetics, minimal systemic toxicity, and prolonged antimicrobial functionality remains a core challenge. Smart-responsive delivery systems—such as pH-, redox-, or enzyme-sensitive nanocarriers—can facilitate on-demand copper ion release tailored to infection-specific microenvironments (Valentino et al., 2024). Integration with multimodal therapeutic platforms that combine photothermal, catalytic, and immunomodulatory actions may further amplify antimicrobial efficacy while minimizing host tissue damage (Wang et al., 2023). Additionally, conjugating copper-based nanoparticles with QS inhibitors, biofilm-dispersal agents (including enzymes, antibiofilm peptides, and small-molecule dispersants), or efflux pump inhibitors offers a promising strategy to circumvent emerging microbial defense mechanisms (Naga et al., 2023; Fleming and Rumbaugh, 2017; Al-Madboly et al., 2024; Allamyradov et al., 2024).

Third, the clinical and industrial translation of copper-based nanotechnologies critically depends on the development of standardized evaluation protocols that encompass antimicrobial efficacy, cytotoxicity, pharmacokinetics, and environmental fate (Gupta et al., 2024; Kent and Vikesland, 2016; Ren et al., 2021; Yao et al., 2018; Gorsse et al., 2023; Ji et al., 2021; de Oliveira Neto et al., 2024). The current lack of harmonized benchmarks not only hinders regulatory approval but also contributes to poor reproducibility across studies. Establishing comprehensive, globally accessible databases on copper nanotoxicology, biocompatibility, and long-term ecological effects will be essential to align academic innovation with policy frameworks and public health imperatives.

Fourth, copper-based nanocarriers are well-positioned to contribute meaningfully to the development of targeted drug delivery systems (de Oliveira Neto et al., 2024). Their intrinsic mesoporous architectures, high surface reactivity, and tunable surface chemistry enable efficient drug loading, selective targeting, and stimuli-responsive release. The integration of photothermal conversion, Fenton-like catalytic activity, and inherent antimicrobial properties makes copper-based nanoparticles uniquely suited for synergistic applications in cancer therapy, wound healing, and infectious disease management (Lv H. et al., 2022; Singh et al., 2025; Tan T. et al., 2024).

Fifth, environmental sustainability must be embedded as a core design principle in copper nanotechnology (Rajput et al., 2020). As concerns rise regarding the accumulation of copper-based nanoparticles in terrestrial and aquatic ecosystems, bioinspired remediation strategies are attracting growing attention (Pande et al., 2022). Fungal, algal, and microbial detoxification systems—particularly those employing adsorption, enzymatic reduction, and biomineralization—offer promising, low-energy solutions to mitigate nanoparticle pollution (Ha et al., 2022). In parallel, circular economy approaches, including recyclable copper composites, green synthesis routes, and controlled-degradation systems, will be essential for minimizing ecological footprints (Kimber et al., 2020; Wahab et al., 2024; Giri et al., 2024).

In summary, the convergence of intelligent material design, bio–nano interface engineering, and ecological stewardship defines a new frontier for copper-based nanotechnologies. As these innovations mature, they hold significant potential to transform medicine, public infrastructure, agriculture, and environmental protection. Strategic investments in interdisciplinary collaboration, regulatory alignment, and sustainable manufacturing will be pivotal in positioning copper nanotechnology as a cornerstone of next-generation antimicrobial and ecological defense systems. Nonetheless, significant limitations persist—including the lack of regulatory harmonization, uncertainties surrounding long-term biosafety, and challenges related to ecological compatibility—which must be systematically addressed to enable their responsible and scalable deployment.
